# A cross-sectional study of sociodemographic factors and their influence on quality of life in medical students at Sao Paulo, Brazil

**DOI:** 10.1371/journal.pone.0180009

**Published:** 2017-07-10

**Authors:** Mario Ivo Serinolli, Marcia Cristina Zago Novaretti

**Affiliations:** Health Administration Graduate Department, Universidade Nove de Julho, Sao Paulo, Sao Paulo, Brazil; Institute of Psychiatry, UNITED KINGDOM

## Abstract

**Background:**

Various sociodemographic factors can affect the quality of life of medical students and interfere in their ability to study. A deeper understanding of these factors may facilitate improvements in learning and retention of medical students.

**Methods:**

We conducted a cross-sectional study of 405 medical students, representing 65.3% of the total student body (years 1–6), at a private medical school in São Paulo, Brazil. Among the entire study group, 177 students (43.7%) were male, and 228 (56.3%) were female. The mean age was 23.55 years (SD = 3.98 years, range: 18–40). The World Health Organization Quality of Life-Biomedical Research and Education Facility (WHOQOL-BREF) scale was used to evaluate the following sociodemographic factors: age, sex, academic year, daily traveling time, housing conditions, smoking, weight, height, participant’s and his/her parents’ education background, students who had a degree or not and religious beliefs. The reliability of the WHOQOL-BREF was evaluated using Cronbach’s analysis, and the association of sociodemographic factors with quality of life was examined using multivariate regression analysis.

**Main Results:**

Quality of life was significantly higher in medical students with religious beliefs (β 0.14 for psychological domain; β 0.11 for environmental domain) when compared with that in those with no religious beliefs. BMI was negative associated with QOL in medical students (β -0.11 for physical domain; β -18.9 for the psychological domain). In both male and female students, longer daily traveling time was negative related to QOL (β -0.11 for environmental domain). Having at least one parent who was a doctor was associated with a better quality of life (β 0.17 for environmental domain). Male students presented with significantly higher mean scores for three of the four domains evaluated (β 0.20 for physical domain; β 0.25 for psychological domain; β 0.14 for social domain).

**Conclusion:**

This study has provided novel insights into the effects of sociodemographic factors, physical traits, and religious beliefs on the quality of life of medical students. These findings may facilitate improvements in physical, psychological, and social support for medical students at a critical stage in their training, thereby providing tools for student better adjustment to medical school.

## Introduction

In the 21st century, advances in medicine and in health technology have provided increased access to medical research databases, social networks, and apps, making it more difficult to be a medical student and ultimately a physician [1]. Medical students experience greater stress compared with university students in other fields due to the long hours of training, stringent academic demands, frequent examinations, and constant exposure to new and often stressful clinical experiences, all of which may lead to anxiety, depression, and burnout [2, 3]. Moreover, the competitive environment persists after medical school and remains a substantial component of a physician’s life. Medical doctors are expected to deliver high-quality health care, possess strong communication skills, collaborate with other physicians, be accountable to the wider medical team, and adopt a patient-centered approach [4]. They function as educators, leaders, team members, managers, and policymakers. To prepare medical students for these challenges, Cruess *et al*. [1] emphasized the need to reframe medical education and to transition from teaching the concept of professionalism to a concept of aligning medical professionalism with the student’s personal identity [4].

Using various assessment tools, several studies demonstrated that medical education and training could negatively affect a student’s physical and mental health, thereby reducing their quality of life (QOL) [5–7]. Furthermore, the QOL of medical students can greatly change during the course of their academic medical training [5, 6, 8]. Recently, Tempski *et al*. [9] demonstrated that strategies designed to improve resilience among medical students might ameliorate the emotional distress associated with medical training. However, the effect of various factors that affect the QOL of the general population, such as obesity [10] and religious beliefs [11], on the QOL of medical students remains unclear.

This study was undertaken to evaluate the effect of various sociodemographic factors on the QOL of medical students, with the goal of improving learning and retention at medical school. We hypothesized that normal body index and religious beliefs are positively correlated with the QOL of medical students. We conducted a cross-sectional study of students at a private medical school in Sao Paulo, Brazil using the World Health Organization Quality of Life-Biomedical Research and Education Facility (WHOQOL-BREF) scale questionnaire [12], a tool that facilitates international cross-cultural comparisons of the QOL [12, 13]. The questionnaire is available in several languages that are spoken in both developed and developing countries [6, 7], and it has proven to be an effective tool to analyze the QOL [12, 13].

## Materials and methods

### Study setting and population

Universidade Nove de Julho is a private University with more than 130,000 students. Among the total student body, 720 students are enrolled at the School of Medicine, with 60 new medical students enrolling each semester. Medical training takes at least 8 hours per day, 5 days a week. The Nove de Julho School of Medicine utilizes an innovative teaching strategy to achieve their mission of preparing future primary care physicians. The curriculum comprises 12 semesters of full-time training with 4 semesters of basic sciences, 2 semesters of module training, and 4 semesters of internship. The students begin interacting with patients in a primary care setting during their first semester. During the first 4 semesters, the students take anatomy, histology, biochemistry, physiology, molecular biology, epidemiology, medical ethics, pathology, pharmacology, embryology, statistics, microbiology, parasitology, scientific projects, and primary care. Each course consists of lectures, seminars, and extensive studying. The next phase of medical school is a 2-year period referred to as the modular step. The modular step consists of training in semiology, internal medicine, pediatrics, surgery, community medicine, gynecology and obstetrics, and an introduction to imaging. In addition, the medical students are encouraged to participate in medical rounds and outpatient consultations under close supervision. The last phase of medical school is the internship, in which students do rotations in pediatrics, internal medicine, surgery, psychiatry, gynecology and obstetrics, emergency care, and primary care. Patients with complex or unique conditions are the focus of interdisciplinary discussion with professors throughout the different phases of training.

### Study design

We conducted a cross-sectional study from October to November 2014. Medical students at Universidade Nove de Julho in their 1st to 12th semesters voluntarily participated in the study. After the study participants provided informed consent to the authors (MIS or MCZN), they received access to an online survey through a link to the Informatics Laboratory of Universidade Nove de Julho, where each participant independently and anonymously completed a questionnaire comprising 45 questions. The students had 15 minutes to complete the questionnaire. The authors were available to clarify any questions during the entire data collection period. We specifically avoided administering the questionnaire around the time of academic assessments to minimize the potential influence of additional academic stress and/or anxiety [14].

### Ethics statement

This study adhered to the guidelines of Resolution 466/12 of the Brazilian National Health Council for experiments with humans. It was approved by the Ethics Committee on Human Research of Universidade Nove de Julho under number 014341 in accordance with the Helsinki Declaration. Each participant was well informed of the content and the aim of the questionnaires. The participants had the option of declining to answer any or all questions if they did not wish to do so. All subjects provided written consent immediately prior to data collection. This was an anonymous survey, and the data obtained remain confidential.

### Questionnaire

The questionnaire was composed of two sections. The first section collected the following sociodemographic data: age, gender, place of birth, place of residence, distance between residence and university, time spent getting to/from the university, weight, height, religious beliefs (no religion, or religion), and information regarding a participant’s and his/her parents’ education background, students who had a degree or not and habits [3, 14, 15]. In the second section of the questionnaire, we administered a Brazilian/Portuguese version [16] of the WHOQOL-BREF, a widely used, generic QOL assessment tool provided by the World Health Organization (WHO) [13] that was validated by Fleck *et al*. in 2006 [17]. QOL assessment is based on answers to questions in four domains: physical health (7 questions), psychological health (6 questions), social relationships (3 questions), and environment (8 questions). Two additional questions assessing general health conditions and QOL satisfaction were included in the second section of the study questionnaire. Each answer is based on self-reported assessments using a 5-point Likert scale ranging from 1 (very dissatisfied/very poor) to 5 (very satisfied/very good). The raw domain scores were transformed into a linear scale between 0 and 100 to facilitate comparisons with another questionnaire used to address QOL (the WHOQOL-100) in accordance with previously published guidelines [18]. Higher scores indicate greater QOL, with the exception of three questions used to assess a negative parameter.

### Statistical analysis

For continuous variables, the data are presented as mean ± standard deviation (SD). For categorical variables, the data are presented as frequencies and percentages, and data associated with non-normally distributed variables are presented as median values. Body mass index (BMI) was classified as overweight (25 < BMI <30), obese (BMI ≥ 30), or normal/underweight (BMI ≤ 24.9). Time to get to/from the university was classified as less than 30 minutes, > 30 < 60 minutes, > 60 < 120 minutes, > 120 < 180 minutes. We calculated Cronbach’s coefficient to determine the internal consistency reliability. We considered Cronbach alpha values over 0.70 as acceptable [19]. To investigate a relationship between WHOQOL-BREF scores and sociodemographic factors, we compared QOL among the study participants using the Student’s t-test and one-way analysis of variance (ANOVA). Multiple regression models were performed. Physical, Psychological, Social and Environmental domains were included as dependent variables. Age, gender, academic year, students’ hometown, BMI, parents background education, students’ prior degree, religion beliefs, smoking, time to get to/from university were analyzed as predictor variables. For each model, the standardized beta coefficient (β), p-value and the explained variance (R2) were calculated. A p-value < 0.05 in the statistical analysis was considered significant. Statistical analyses were conducted using the SAS statistical software (version 9.3, SAS Institute, Cary, NC).

## Results

### Socio-demographic characteristics of the study population

The study population comprised 405 medical students in their 1st through 12th semesters, representing 65.3% of the medical students at Universidade Nove de Julho. The study group included 177 (43.70%) males and 228 (56.30%) females. The mean age was 23.55 years (SD = 3.98, range: 18–40 years). There was no statistically significant difference in age between the male and female participants (p = 0.9379). An overview of sociodemographic characteristics of the study population is presented in Table 1.

**Table 1:** Sociodemographic characteristics of the study population.

Characteristics	N (%)	Mean	SD*	Minimum	Maximum
**Age (years)**					
**male**	**177 (43.70)**	**23.57**	**4.13**	**18**	**40**
**female**	**228 (56.30)**	**23.54**	**3.87**	**18**	**40**
**total**	**405 (100.00)**	**23.55**	**3.98**	**18**	**40**
**Weight (Kg)**					
**male**	**177 (43.70)**	**82.44**	**13.59**	**57**	**139**
**female**	**228 (56.30)**	**60.76**	**11.22**	**43**	**115**
**total**	**405 (100.00)**	**70.24**	**16.34**	**43**	**139**
**Height (cm)**					
**male**	**177 (43.70)**	**177.90**	**5.87**	**162**	**197**
**female**	**228 (56.30)**	**164.20**	**6.30**	**150**	**179**
**total**	**405 (100.00)**	**170.19**	**9.14**	**150**	**197**

* SD: standard deviation

### Reliability

The Cronbach’s alpha coefficient of the complete WHOQOL-BREF questionnaire was 0.8998. The coefficients of the physical health, psychological health, social relations, and environmental domain were 0.7201, 0.7664, 0.7152, and 0.7415, respectively.

### QOL according to academic year and gender

The number of years in medical school was not significantly associated with QOL (Table 2). The highest mean WHOQOL-BREF QOL scores were observed in the social relations domain, whereas the lowest mean scores were observed in the environmental domain. Significantly higher mean scores for three out of the four domains evaluated were observed in males (physical health, p = 0.0035; psychological health, p = 0.0005; social relations, p = 0.0161). There was no significant difference between males and females with respect to the environmental domain of the questionnaire (p = 0.9832) (Table 2). After multiple separate regression analysis we also found that female gender was related to QOL in all domains, except for the environmental one. There was a moderate effect of gender in physical (**β** .20) and psychological (**β** .26) domains and a weak effect on social domain (**β** .15) (Table 3).

**Table 2:** QOL domain scores (mean and standard deviation) according to academic year and gender.

Academic Year	N (%)	Physical Health (mean and standard deviation)	Psychological Health (mean and standard deviation)	Social Relations (mean and standard deviation)	Environmental (mean and standard deviation)
**1st**	**86 (21.23)**	**65.03±13.53**	**65.89±15.11**	**71.32±18.85**	**59.41±13.71**
**male**	**48 (55.81)**	66.66**±13.57**	67.62**±15.57**	74.47**±16.07**	59.04**±12.08**
**female**	**38 (44.19)**	62.96**±13.37**	63.70**±14.43**	67.32**±21.43**	59.86**±15.69**
**2nd**	**102 (25.19)**	**61.97±15.33**	**64.82±14.00**	**70.42±20.63**	**57.01±14.95**
**male**	40 (39.22)	64.37**±14.98**	67.81**±12.62**	72.70**±17.29**	55.00**±14.37**
**female**	62 (60.78)	60.42**±15.47**	62.90**±14.60**	68.95**±22.54**	58.31**±15.28**
**3rd**	**97 (23.95)**	**63.84±15.09**	**65.08±16.02**	**72.94±17.55**	**59.73±15.42**
**male**	38 (39.18)	65.31**±17.13**	66.99**±18.89**	73.02**±20.63**	60.44**±16.91**
**female**	59 (60.82)	62.89**±13.68**	63.84**±13.91**	72.88**±15.44**	59.26**±14.51**
**4th**	**59 (14.57)**	**61.62±12.95**	**62.50±16.59**	**69.20±23.37**	**60.96±15.57**
**male**	27 (45.76)	65.21**±13.71**	65.43**±16.65**	73.76**±20.24**	60.76**±14.11**
**female**	32 (54.24)	58.59**±11.64**	60.02**±16.39**	65.36**±25.40**	61.13**±16.93**
**5th**	**35 (8.64)**	**65.40±15.53**	**68.69±16.24**	**73.09±17.97**	**60.08±16.84**
**male**	14 (40.00)	71.68**±17.06**	79.46**±9.45**	76.19**±21.39**	62.50**±22.02**
**female**	21 (60.00)	61.22**±13.23**	61.50**±15.97**	71.03**±15.50**	58.48**±12.66**
**6th**	**26 (6.42)**	**67.54±17.09**	**64.90±15.68**	**65.71±19.76**	**56.85±16.97**
**male**	10 (38.46)	67.50**±19.00**	67.08**±17.50**	71.66**±10.54**	59.37**±13.50**
**female**	16 (61.54)	67.41**±16.43**	63.54**±14.86**	61.97**±23.36**	55.27**±19.08**
**TOTAL**	**405 (100.0)**				
**male**	177 (43.70)	66.08±15.23	68.10±15.81	73.63±18.05	58.98±15.00
**female**	228 (56.30)	61.79±14.06	62.79±14.70	68.89±20.68	59.01±15.36
**p-test**		0.0035	0.0005	0.0161	0.9832
**test used**		t-student	t-student	t-student	t-student

**Table 3:** Associations between quality of life of medical students and predictor variables.

Variable	Physical QoL		Psychological QoL		Social QoL		Environmental	
	β *	p	β	p	β	p	β	p
**Academic year**	.0522533	**0.305**	.0180772	0.716	-.0148977	0.770	.0042333	0.932
**Gender**	.2024125	0.000	.2598076	0.000	.1474219	0.007	.0140499	0.792
**At least one parent physician**	-.0070457	0.889	.054317	0.271	.0475324	0.348	.1752481	0.000
**Religion beliefs**	.0683935	0.174	.1489346	0.002	.0702851	0.162	.1111119	0.025
**Time to get to /from**	-.0812605	0.151	-.0418517	0.448	-.1227917	0.030	-.1883522	0.001
**Smoking**	-.0296317	0.553	-.0245209	0.615	.016941	0.735	-.0040716	0.934
**Previous degree**	.0093911	0.851	.0355388	0.466	-.0368284	0.462	-.0755587	0.124
**Hometown**	-.0504292	0.374	-.0680633	0.219	-.0267316	0.638	-.1156196	0.038
**BMI**	-.1118018	0.038	-.1872911	0.000	-.0878492	0.103	-.0610303	0.248
**R2**	4.6%		9.3%		4.5%		8.1%	

QOL = quality of life;

*β = indicates standardized β coefficient

### QOL according to sociodemographic factors

In this study, we evaluated the effect of sociodemographic factors on the QOL of medical students in Sao Paulo, Brazil. In this study, we found no statistical difference between students who had previously graduated compared with those who had not, or smokers compared with non-smokers. Nearly 67% of the study participants were from outside Sao Paulo. At first, we did not observe statistically significant differences in QOL scores in students living in Sao Paulo compared with students living outside of Sao Paulo, however we found a negative weak effect on the environmental domain (β -0.12) after multiple regression analysis (Table 3).

Higher mean psychological health scores were observed in students who held religious beliefs (p = 0.0068). In addition, participants with at least one parent who held a medical degree had higher mean scores in the environmental domain (p = 0.0004). A commute between home and university longer than two hours had a significant negative impact on the QOL with respect to the social relations and environmental domain (p = 0.0301 and p = 0.0299, respectively). Finally, a BMI ≥ 30 was associated with lower QOL scores in the psychological, social relations, and the physical health domain, but not in the environmental domain (Table 4).

**Table 4:** QOL scores (mean and standard deviation) of medical students according to sociodemographic factors.

Academic Year	N (%)	Physical Health (mean and standard deviation)	Psychological Health (mean and standard deviation)	Social Relations (mean and standard deviation)	Environmental (mean and standard deviation)
**Hometown**					
**São Paulo**	134 (33.09)	63.51±15.18	65.60±16.75	69.52±20.23	59.30±16.35
**Other city**	271 (66.91)	63.74±14.51	64.86±14.72	71.67±19.42	58.85±14.60
**Religious beliefs**					
**Yes**	315 (77.78)	64.10±14.81	**66.21**±14.75*	71.50±19.93	59.76±14.62
**No**	90 (22.22)	62.14±14.39	**61.25**±17.03	69.07±20.91	56.35±16.84
**Previous Graduation**					
**Yes**	59 (14.57)	63.74±15.83	66.10±15.52	68.50±19.39	56.14±14.09
**No**	346 (85.43)	63.65±14.55	64.94±15.40	71.39±19.74	59.49±15.33
**At least one parent physician**					
**Yes**	88 (21.73)	64.24±18.26	67.23±16.42	73.10±20.12	**64.02**±16.87**
**No**	317 (78.27)	63.50±13.61	64.52±15.08	70.37±19.56	**57.61**±14.40
**Time to get to/from university**					
**< 30 min**	225 (55.56)	63.89±14.18	64.98±14.94	**73.07**±18.90***	60.72 (13.86)****
**30–60 min**	83 (20.49)	65.36±14.80	65.76±14.48	**68.57**±20.59	**58.62**±17.40
**60–120 min**	56 (13.83)	63.84±15.77	68.38±15.38	**71.13**±19.20	**56.14**±13.60
**120–180 min**	41 (10.12)	59.80±15.59	60.06±18.73	**64.02**±21.36	**54.27**±18.11
**Smoking**					
**Yes**	47 (11.60)	62.53±13.04	63.65±14.53	71.27±20.61	57.51±14.60
**No**	358 (88.40)	63.81±14.94	65.30±15.52	70.92±19.60	59.20±15.27
**BMI**					
**18.5–24.9**	273 (67.41)	**63.30**±14.36^#^	**65.64**±14.74^##^	**74.00**±20.12^###^	59.39±15.35
**25–29.9**	93 (22.96)	**67.97**±14.32	**66.80**±15.52	**73.66**±18.07	60.18±13.78
**≥30**	39 (9.63)	**55.95**±14.98	**57.37**±17.74	**64.32**±19.30	53.53±16.46

*p = 0.0068 (t-test),

**p = 0.0004 (t-test),

***p = 0.0301 (one-way ANOVA),

****p = 0.0299 (one-way ANOVA),

^#^p = 0.0001 (ANOVA),

^##^p = 0.0034 (ANOVA),

^###^p = 0.0451 (ANOVA)

Separate multiple linear regression analyses were performed to identify predictors variables for the physical, psychological, social and environmental QOL, as measured by the WHOQOL-BREF (Table 3). In our study, although less than 10% of the variance in all domains evaluated was explained, we found some significant statistical p-value in the models. We found that two variables, gender (**β** .20) and BMI (**β** -.11) are the strongest predictors in the physical domain. For the psychological domain, gender (β 0.26) and BMI (β -.19) were the strongest predictors. Time spent to /from the university was negative associated to the social domain (β -.12). The strongest predictors in the environmental domain were time spent to /from the university (β -.19) and students parent with a medical degree (β.17).

Student´s parent with medical doctor degree was perceived as a positive predictor of QOL in the environmental domain. This result can be partially, explained by the fact that parents with a medical degree could influence their offspring for a better understanding of the medical school environment.

Religion beliefs were positive related to psychological and environmental domains, while time to get to to/from the university was identified as negative related to psychological and environmental domains. Students hometown out of São Paulo, where the medical school campus is located, was negative associated with environmental domain of QOL as well. Finally, BMI was negative related to QOL in physical and psychological domains.

## Discussion

This study was conducted to identify sociodemographic factors that potentially influence the QOL of medical students attending a private medical school in Sao Paulo, Brazil. Consistent with our initial hypothesis, we found that a normal BMI and religious beliefs were associated with greater QOL in medical students. In addition, we observed lower scores in the physical, social, and psychological domains of the QOL questionnaire in female students compared with male students.

### Obesity

The negative impact of obesity on the QOL in the general population has previously been described elsewhere [20–22]. The increasing prevalence of obesity worldwide is attributed to lifestyle changes, and this phenomenon is associated with an increased incidence of chronic diseases and mental disorders [20, 21]. The WHO estimated that approximately 13% of the world´s adult population were obese and 39% were overweight in 2013. If current trends continue, approximately 57.8% of the adult population will be classified as overweight/obese by 2030 [23]. Malta *et al*. [24] reported that an alarming 51.0% of Brazilian adults were overweight and 17.4% were obese in 2012, with an annual increase in these rates of 1.37% and 0.89%, respectively.

The proportion of medical students that are overweight ranges from 19.7% in American freshmen [25] to 39.3% in Greek students [26]. Obesity was reported in 4.8% of American medical students [25] and in 10.2% of Pakistani medical students [27]. Furthermore, one study reported that the rates of obesity/overweight increased from 24.9% in the first year of medical school to 37.1% in the last year (p < 0.005) [28].

In this study, the rates of overweight (23.0%) and obesity (9.6%) were consistent with previous reports. Although a previous study evaluating Brazilian medical students attending a private university observed lower rates of obesity and overweight (1.4% and 16.4%, respectively), the study was conducted in 2006 [29].

Due to its reported impact on the QOL in the general population [20–22], we analyzed the potential influence of obesity in the QOL of medical students. Our findings confirmed our hypothesis that obese medical students have a lower QOL compared with nonobese students. Lower QOL scores were observed in obese students with respect to two of the four domains evaluated (physical and psychological).

These results may reflect, in part, the discrimination, lack of acceptance, or social stigma associated with overweight and obese individuals [30].

### Religious beliefs

Religious beliefs and spirituality are reported in nearly 90% of the population worldwide [31]. The impact of spirituality and religious beliefs on the QOL, especially with respect to mental health, have been previously reported [32, 33]. Several studies concluded that religious beliefs reduce the incidence of anxiety, depression, drug addiction, and suicide attempts [31–33]. Religious beliefs are also associated with attitudes toward clinical interactions, assisted reproductive technologies, life and death concepts, and legalized abortion [34].

A large cross-cultural study described the positive effect of spirituality, religion, and personal beliefs on components of the QOL [32]. The authors observed the strongest correlations with the psychological and social domains.

Although physicians need to deal with patient expectations and personal beliefs, there is a scarcity of data on the religious beliefs of physicians. Recently, Kim and Roth reported that the incidence of depression was greater in Korean medical students without religious beliefs compared with religious students [35].

A large Brazilian study reported that 33.9% of medical students did not report a religious affiliation and that religious beliefs influence opinions on controversial medical ethics issues [36]. A study of 275 medical students in New Zealand found that 42.5% of the study participants had no religion affiliation [37].

Compared with previous studies, we observed a lower percentage of medical students with no religious beliefs (22.2%). This difference might reflect the study population, as the research conducted by Lucchetti *et al*. included public, private, rural, and cosmopolitan universities, thereby representing the heterogeneity of the Brazilian society [36].

In the present study, we identified an association between religious beliefs and improved QOL with respect to psychological health (β .15; p = 0.002) and to environmental domain (β .11; p = 0.0025). By contrast, Henning *et al*. [37] did not observe a similar effect in medical students in New Zealand.

### The effect of academic year on the QOL

Several publications have already reported the effect of academic year on the QOL in medical students [38–40]. In this study, we did not observe any effect of academic year on the QOL. This finding might be partially explained by the fact that Universidade Nove de Julho subscribes to the philosophy of empowerment education, as first described by Paulo Freire [41]. Freire proposes that empowerment education promotes health in all aspects of life. His model suggests that active participation in group activities and dialogue enhances the ability to change the education process and to change one’s life [41]. Students have free access to professors, coordinators, and the dean of the School of Medicine. In addition, the university makes efforts to periodically engage students in an open dialogue during regularly scheduled meetings. In these meetings, the students provide their own insights into approaches that might improve the quality of their medical education and their communication skills.

Zhang *et al*. [6] reported significant differences in the QOL according to academic year, particularly with respect to the psychological and physical domains [4]. Domantay [2] described an association of some academic years with lower QOL in medical students. He attributed this effect to the specific challenges that arise during different academic years, such as adaptation during the first year and clinical clerkships during later years [2].

### Gender effect

In previous centuries, medical students were predominantly male. In 2006, the WHO reported that the number of females attending medical school was increasing worldwide [42]. In the United States (US), women account for 47% of medical students; however, only 21% of full-time professors are women, indicating that this is still a relatively new trend [43]. Nevertheless, in some countries, including Great Britain, China, and Brazil, women have been outnumbering men in medical sciences in recent years [6, 44,, 45]. This is a relatively new global phenomenon, and, although it has been gaining attention, the consequences of this shift remain unclear.

In 2012, a study of 150 Pakistani medical students reported that females had higher levels of anxiety. The authors attributed this observation to greater emotional vulnerability in female students, due in part to a patriarchal society that discourages women from pursuing higher education [46]. However, this explanation might not be relevant to the results described in our study as the Brazilian society is more liberal, and it promotes equal opportunities for men and women.

A systematic review of 40 studies [47] revealed that female medical students in the US and Canada experienced more personal distress. However, it was unclear if this finding was directly associated with medical education, and the authors emphasized that their results required further investigation.

Another recent systemic review reported that females are more prone to develop anxiety before or after academic assessments [14]. However, this type of stress/anxiety cannot explain our results, as we avoided collecting data around the time of academic assessments.

Paro [48] and Domantay [2] also reported lower QOL scores for female medical students [2, 3]. Furthermore, several authors [6, 8, 49] reported lower QOL with respect to psychological and physical domains of the WHOQOL-BREF questionnaire in female medical students.

In our study, we observed lower QOL scores in the psychological, physical, and social relation domains in female medical students. These findings are consistent with a culture that encourages men to appear less emotional and to hide their feelings and weaknesses. However, additional research is required to address this hypothesis.

### Limitations

Several limitations to our study must be noted. The results are limited to a single medical school in a developing country in one of the largest cities in the world. Consequently, external factors that might affect the QOL of the general population, such as heavy traffic and a high rate of violence, might also influence the participants of this study. Despite these limitations, the compelling data observed in this study might provide a basis for research in other localities.

In addition, strategies to help overcome the challenges associated with poor QOL identified in this study can be implemented. For example, promoting social activities and providing tutoring services might help overcome the observed differences between males and females. In addition, making healthy foods available and providing health services might reduce obesity rates.

The stress associated with a long commute between home and university might be alleviated by creating a department that helps students living in areas outside Sao Paulo find housing closer to the university.

### Conclusion

This study has provided a deeper understanding of the effects of sociodemographic factors, physical traits, and religious beliefs on the QOL in medical students. These findings might inform strategies to improve physical, psychological, and social support for medical students at a critical stage in their training, thereby providing tools to a better adjustment to medical schools.

## Supporting information

S1 File The all quality of life of medical students results. (XLSX)

S2 File History of anxiety, panic attacks or depression and analysis of the impact on quality of life in medical students. (PDF)

S3 File Impact of physical activity practice on quality of life of medical students, Nove de Julho University (Uninove). (PDF)
