# Supplementary material for: A cross-sectional study of sociodemographic factors and their influence on quality of life in medical students at Sao Paulo, Brazil
Source: PLoS One. 2017 Jul 10;12(7):e0180009. doi: 10.1371/journal.pone.0180009 (PMC5503183; doi:10.1371/journal.pone.0180009)
Supplement: S2 File — (PDF) [file pone.0180009.s002.pdf]

REVISTA DE GESTÃO EM SISTEMAS DE SAÚDE

E-ISSN: 2316-3712
DOI: 10.5585/rgss.v4i2.205
Data de recebimento: 28/08/2015
Data de Aceite: 29/10/2015
Organização: Comitê Científico Interinstitucional
Editor Científico: Marcia Cristina Zago Novaretti
Editora Adjunta: Simone Aquino
Avaliação: Double Blind Review pelo SEER/OJS
Revisão: Gramatical, normativa e de formatação

ANTECEDENTE DE ANSIEDADE, SÍNDROME DO PÂNICO OU DEPRESSÃO E ANÁLISE DO IMPACTO NA QUALIDADE DE VIDA EM ESTUDANTES DE MEDICINA

RESUMO

Alta competição por resultados, muitas horas de estudo, carga horária acadêmica elevada, entre outros motivos, são apontadas como causa do elevado índice de ansiedade, pânico e depressão nos estudantes de Medicina. Avalia-se o impacto do histórico diagnosticado pelo médico dessas patologias na qualidade de vida dos estudantes de Medicina por meio de pesquisa transversal com aplicação do questionário da Organização Mundial da Saúde para avaliação da qualidade de vida – formato curto (WHOQOL-BREF), acrescido de um questionário com perguntas relacionadas a histórico de ansiedade, síndrome do pânico ou depressão anterior, religião e onde residem seus familiares em 405 alunos de Medicina da cidade de São Paulo, Brasil. Nos resultados, os alunos com histórico prévio de ansiedade, pânico ou depressão (34,57%) obtiveram pior índice de qualidade de vida demonstrado nos domínios físico, psicológico, relação social e ambiental, quando comparados ao grupo sem histórico prévio de depressão ($p < 0,005$). Alunos que relatam ter religião têm melhor qualidade de vida, com índices superiores no domínio psicológico ($p < 0,005$). Estudantes cujas famílias residem em outras cidades têm histórico mais elevado de ansiedade, síndrome do pânico ou depressão (teste exato de Fisher = 0.014). Conclusão: neste trabalho, história de ansiedade, pânico ou depressão e alunos sem religião apresentam piores índices de qualidade de vida e devem ser monitorados por programas especiais para melhorar o bem-estar, com ênfase para os alunos provenientes de outras cidades.

Palavras-chave: qualidade de vida; estudantes de medicina; estresse; ansiedade; pânico; depressão; qualidade; religião; formação médica.

HISTORY OF ANXIETY, PANIC ATTACKS OR DEPRESSION AND ANALYSIS OF THE IMPACT ON QUALITY OF LIFE IN MEDICAL STUDENTS.

ABSTRACT

High competition for results, too many study hours, courses with a huge hours total, among other reasons, are pointed out as causes for the high rate of anxiety and depression in medical students. This work aims to evaluate the impact of previous doctor-diagnosed history of anxiety, panic syndrome or depression on quality of life of medical students and religion through a cross-section study with application of the World Health Organization questionnaire to assess quality of life in its short format (WHOQOL-BREF), plus a questionnaire with questions related to the history of previous anxiety, panic syndrome or depression, religion and where their families reside in 405 medical students from São Paulo, Brazil. The results showed that students with prior history of anxiety, panic syndrome or depression diagnosed by doctors (34.57%) had lower quality of life, an index demonstrated in the four domains: physical, psychological, social and environmental, when compared to the group without prior history of depression ($p <0.005$). Students who have religion have better quality of life with higher rates in the psychological domain ($p <0.005$). Students whose families live in other cities have a higher history of depression (Fisher exact test = 0.014). Conclusion: students who have a history of diagnosed anxiety, panic syndrome or depression and have no religion show the worst indices of quality of life and should be monitored by support programmes during medical school, with emphasis on students whose families live in other cities. Special programmes to improve the well-being should be studied and implemented.

Keywords: quality of life, medical students, stress, anxiety, depression, quality, panic syndrome, religion, medical training.

Mario Ivo Serinolli[1]
Maria da Penha Monteiro Oliva[2]
Elias El-Mafarjeh[3]

[1] Doutor em Medicina pela Faculdade de Medicina da Universidade de São Paulo – USP, Brasil
Professor de Pós-Graduação do Mestrado Profissional em Gestão e Sistemas de Saúde da Universidade Nove de Julho, Brasil
E-mail: mserinolli@gmail.com

[2] Mestranda em Gestão e Sistemas de Saúde da Universidade Nove de Julho – UNINOVE, Brasil
E-mail: mpoliva@uninove.br

[3] Graduando pela Faculdade de Medicina da Universidade Nove de Julho – UNINOVE, Brasil
E-mail: alshafing@gmail.com

1. **INTRODUÇÃO**

O curso de Medicina é mundialmente conhecido pela sua complexidade e dificuldade devidas à grande exigência para os alunos entrarem na faculdade, por questões socioeconômicas e pelo tempo prolongado de estudo, sacrifícios, distanciamento da família e dos amigos, bem como pela necessidade de grande resistência física e emocional (Cataldo Neto, Cavalet, Bruxel, Kappes, & Silva, 1998; De Marco, Rossi, & Millan, 1999; Villanueva & Haivas, 2006).

A relação entre o estresse e a Medicina é estudada em diversos países, principalmente seu impacto na qualidade de vida do estudante de graduação (Ludwig et al., 2015; Maria Inês, 2009; Mosley et al., 1994; Rossi, De Marco, & Millan, 1991). Pesquisas indicam que a ocorrência de transtornos mentais e depressão aumentam no decorrer do curso médico (Brazeau et al., 2014; Brazeau, Schroeder, Rovi, & Boyd, 2010; Dyrbye, Thomas, & Shanafelt, 2005; Gold, Johnson, Leydon, Rohrbaugh, & Wilkins, 2015; Sullivan & Buske, 1998).

A depressão causa grande sofrimento psíquico e diminuição no rendimento acadêmico, com impacto na vida profissional futura dos estudantes de medicina (Hojat et al., 1993). No entanto, há necessidade de mais explorações para se avaliar o real impacto na vida acadêmica e futura do médico (Dyrbye, Thomas, & Shanafelt, 2006).

Essa tem sido uma das preocupações em função da grande necessidade de formar médicos para nosso país com características compatíveis com o setor de assistência médica pública, em que as questões do humanismo e da capacidade de estabelecer vínculo de confiança com os pacientes e familiares são centrais no modelo de atenção à saúde (Brazeau et al., 2010). Complementar o modelo de educação tecnológico e científico com o modelo ético-humanista é o grande desafio a ser superado com enfoque na integralidade das ações de saúde e atenção médica (Araújo, Gomes de Miranda, & Brasil, 2014).

Pesquisas realizadas no Brasil demonstram que a prevalência de depressão entre os estudantes de medicina pode chegar até 28,8% (Paula et al., 2014), com relatos de taxas de adoecimento para alguns transtornos psiquiátricos em estudantes universitários em geral que variam de 15% a 25% (Cavestro & Rocha, 2006; Cerchiari, Caetano, & Faccenda, 2005). Publicações internacionais demonstram prevalências entre 5,7% e 10,6% em estudantes ingleses (Quince, Wood, Parker, & Benson, 2012), chegando a 14,3% de depressão dos alunos da University of Michigan Medical School, nos Estados Unidos (Schwenk, Davis, & Wimsatt, 2010).

Fatores de risco têm sido associados à maior prevalência de depressão, tais como: sexo feminino, terceiro e quarto anos da faculdade, morar sozinho, dificuldades financeiras, falta de atividade física e obesidade (Phelan et al., 2015). Habitualmente, os estudantes com diagnóstico de depressão apresentam baixa autoestima, pior desempenho escolar, isolamento, menor probabilidade de possuir namorado(a) (Kim & Roh, 2014; Roh, Jeon, Kim, Han, & Hahm, 2010).

Investigações realizadas com estudantes de Medicina da Yale School of Medicine, nos Estados Unidos, referem que 19% dos estudantes recebem suporte de saúde mental, sendo que 25% dos estudantes admitiram que, após o início do curso médico, sentem maior necessidade de apoio ou suporte em saúde mental (Gold et al., 2015).

Diversos autores reportam que a prevalência de depressão em estudantes de Medicina está acima das estimativas de índices documentados na população em geral (Kumar, Jain, & Hegde, 2012; Roberts et al., 2001; Zoccolillo, Murphy, & Wetzel, 1986).

Uma pesquisa da OMS sobre a prevalência de depressão na população em geral realizada em dez países, envolvendo 37.000 pessoas, mostra que, em média, o índice de pelo menos um episódio depressivo maior durante a vida variou de 3% no Japão a 16,9% nos EUA, sendo que, na maioria dos países, variou de 8% a 12% (Japão 3,0%, Turquia 6,7%, República Tcheca 7,8%, México 8,1%, Canadá 8,3%, Alemanha 11,5%, Brasil 12,6%, Holanda 15,7%, EUA 16,9% (Andrade et al., 2003).

A depressão é doença que leva à incapacitação e à diminuição dos níveis de produtividade, sendo apontada como causa importante de perda financeira no mundo moderno e grande custo para o sistema de saúde (Donohue & Pincus, 2007). Essa situação gerou o termo "presenteísmo", quando o profissional ou estudante está presente, porém não consegue desenvolver todo o seu potencial e produtividade.

A depressão, de modo geral, é altamente prevalente no mundo e há indícios de que esteja aumentando sua prevalência (Andrade et al., 2003). Trata-se de condição com explicação multicausal, incluindo fatores genéticos, alterações epigenéticas e na disponibilidade de neurotransmissores (serotonina, dopamina, noradrenalina), alterações anatômicas cerebrais no hipocampo, celulares, fatores sociais e psicológicos. A depressão maior não tratada desarranja o funcionamento normal do cérebro e está associada a altos índices de comorbidades psiquiátricas, incluindo distúrbios de ansiedade (ansiedade generalizada; fobias; síndrome do pânico; sintomas somáticos com palpitação, falta de ar, parestesias, gastrointestinais, entre outros; episódios

de ansiedade pós-traumática; episódios de ansiedade aguda) e clínicas (maior incidência de doenças cardiovasculares, doenças autoimunes, entre outras) (Andersson et al., 2015; Kessler et al., 2011; Krishnan, 2015; Lyness, 2015).

O histórico pessoal ou familiar de depressão compõe grupo de risco para apresentar grave quadro depressivo, constituindo-se em fator de proteção ter realizado graduação prévia (Honney, Buszewicz, Coppola, & Griffin, 2010). O sexo feminino, histórico de depressão, a não participação em atividades sociais, as dificuldades de relacionamento e a falta de atividades de lazer foram associados à maior chance de quadros depressivos (Guerrero López et al., 2013; Kim & Roh, 2014; Pucci, Rech, Fermino, & Reis, 2012; Roh et al., 2010).

Também é citado que, quanto maior o requisito de entrada em determinada faculdade, também é maior o risco de desenvolvimento de quadros depressivos, o que poderia ser correlacionado ao vestibular no Brasil. Parece haver maior resistência em procurar ajuda de tutores ou profissionais pelo medo do estigma que essa doença, a depressão, carrega socialmente (Cheng, Poon, Nguyen, & Woodman, 2013; Vankar & Prabhakaran, 2014).

O receio de procurar ajuda caracteriza uma barreira à detecção mais acurada dos fatores de risco, do diagnóstico e tratamento dos casos de depressão (Martin, 2010). Há temor demonstrado entre os deprimidos de ficar apontado entre os colegas como incapaz de ocupar uma vaga de residência ou de prosseguir na carreira, ou mesmo de ser tachado como indivíduo frágil (Wimsatt, Schwenk, & Sen, 2015).

O estigma faz com que o estudante com depressão evite procurar ajuda, até por medo de que o problema fique registrado em seu histórico médico. Muitas vezes, o conhecimento médico do estudante sobre depressão não permite o autodiagnóstico, estando o atraso no diagnóstico relacionado a perdas financeiras pessoais e institucionais (Sharac, Mccrone, & Clement, 2010).

Esse comportamento é mais acentuado entre os homens e se modifica nos diferentes anos da faculdade (Schwenk et al., 2010). Nos estudantes de Medicina, há maior risco de abuso de drogas, álcool e automedicação com antidepressivos, fatos resultantes da questão paradoxal de estarem dentro do serviço de saúde, mas, em função de resistência própria, ou de não terem consciência de seu diagnóstico, ou, ainda, do receio de serem discriminados pelos preceptores médicos ou médicos assistentes ou tutores, não procurarem a atenção médica necessária (Wallace, 2010).

Há distúrbios de ansiedade, consumo de drogas, depressão e até mesmo casos de suicídio em maior número em estudantes e profissionais médicos do que na população em geral – reflexo das condições a que os médicos e estudantes são submetidos em suas atividades (Devi, 2011; Sansone & Sansone, 2009).

Segundo a Fundação Americana para a Prevenção do Suicídio (American Foundation for Suicide Prevention), 300 a 400 médicos cometem suicídio anualmente, uma média de um caso por dia. Há maior risco para os médicos residentes, com maior incidência de doenças mentais graves em virtude da privação do sono, incertezas quanto ao futuro, pouca assistência disponível, isolamento, necessidade de conquistar seu espaço, grande competição e pouco espaço para discutir os insucessos (Downs et al., 2014; Goldman, Shah, & Bernstein, 2015; Rubin, 2014).

Pesquisa realizada pela Canadian Medical Association avaliou 3.520 profissionais de saúde e observou que as principais causas desse perfil insatisfatório são a elevada carga de trabalho, os impactos negativos na vida familiar e pessoal, a escolha da profissão e insatisfação com a área de escolha (Sullivan & Buske, 1998).

No Brasil, recentemente, o Conselho Federal de Medicina publicou artigo sobre a qualidade de vida do profissional no país, em que foi apontado que 5% dos médicos se sentem sem esperança e apresentam pensamentos suicidas (Conselho Federal de Medicina, 2007). Com isso, pode-se notar que as condições estressantes do trabalho para médicos e estudantes não é exclusividade de qualquer país.

Inquérito realizado em Faculdades de Medicina da Malásia descreve que o risco de suicídio entre os estudantes é de 7%. Participaram da pesquisa 537 estudantes de uma faculdade com 625 alunos. Foi aplicado o questionário SBQ-R (Suicidal Behaviours Questionnaire – Revised), que contém quatro itens, a saber: ideia ou tentativa anterior de suicídio, frequência de ideação suicida no último ano, receio de atentar contra a própria vida e se procuraria ajuda no caso de ideação ou tentativa de suicídio. Os fatores correlacionados ao resultado foram presença de quadro depressivo, quebra de relacionamento amoroso, incerteza quanto ao futuro e perda de um bem material valioso que foi roubado ou perdido (Tan, Sherina, Rampal, & Normala, 2015).

Distúrbios da ansiedade e depressão são condições de alta prevalência e frequentemente coexistem, sendo de maior gravidade do que presentes, cada uma, isoladamente. A depressão ansiosa é um subtipo de depressão (Ameringen, 2015). A prevalência de distúrbios da ansiedade e de depressão nos EUA é de respectivamente 28,8% e 16,6% durante o período de vida (Kessler et al., 2005).

Pesquisas demonstram que o distúrbio de ansiedade mais relacionado à depressão é o distúrbio de ansiedade generalizado e os menos

frequentemente associados são a agorafobia e fobias (Alonso, Lépine, & ESEMeD/MHEDEA 2000 Scientific Committee, 2007). Em análise realizada com 1783 pessoas, 75% daquelas com diagnóstico de depressão tinham critérios para distúrbios da ansiedade e 79% daquelas com diagnóstico de distúrbios da ansiedade possuíam critérios para depressão maior durante a vida (Lamers et al., 2011).

A associação de Síndrome do Pânico a Depressão variou entre 14,4% e 42,3% (Gabilondo et al., 2010; Magee, Eaton, Wittchen, McGonagle, & Kessler, 1996). Investigação com 915 mulheres entre 42 e 52 anos encontrou: 10,7% com história de distúrbios da ansiedade e depressão; 53% não reportaram nenhum distúrbio; 22,1% tiveram história de depressão isolada; e 13,8% tiveram distúrbios da ansiedade de forma isolada (Cyranowski et al., 2012).

A depressão maior unipolar, segundo o Diagnostic and Statistical Manual of Mental Disorders, Fifth Edition (DSM-5), da Associação Americana de Psiquiatria, é caracterizada por história de um ou mais episódios depressivos maiores e sem história de mania ou hipomania. Um episódio depressivo principal manifesta-se com cinco ou mais dos seguintes sintomas durante, pelo menos, duas semanas consecutivas; pelo menos um sintoma deve ser ou humor deprimido ou perda de interesse ou prazer: humor deprimido na maior parte do dia, quase todos os dias; perda de interesse ou prazer na maioria ou em todas as atividades, quase todos os dias; insônia ou hipersonia quase todos os dias; perda de peso significativa ou ganho de peso, ou diminuição ou aumento do apetite quase todos os dias; retardo ou agitação psicomotora quase todos os dias observável por outra pessoa; fadiga ou baixa energia, quase todos os dias; diminuição da capacidade de concentração, pensar ou tomar decisões, quase todos os dias; pensamentos de inutilidade ou culpa excessiva ou inapropriada, quase todos os dias; pensamentos recorrentes de morte ou ideação suicida, ou uma tentativa de suicídio (American Psychiatric Association, 2013).

A depressão persistente ou crônica agora engloba a distimia e a depressão unipolar crônica, sendo os critérios a duração de pelo menos dois anos com três ou mais dos seguintes sintomas durante, pelo menos, dois anos consecutivos, e pelo menos um sintoma deve ser humor deprimido: humor deprimido na maior parte do dia, mais dias do que não; diminuição ou aumento do apetite; insônia ou hipersonia; baixa energia ou fadiga; baixa autoestima; dificuldade de concentração ou tomada de decisão; desesperança. Assim, os sintomas não são tão numerosos quanto na depressão maior. Períodos livres de sintomas durante o curso do transtorno depressivo persistente podem ocorrer, mas não podem exceder dois meses consecutivos durante o período de dois anos (ou mais). Transtorno depressivo persistente causa sofrimento significativo ou prejuízo psicossocial. O efeito sobre o funcionamento social e ocupacional varia, mas pode exceder o da depressão maior (Lyness, 2015).

Há vários subtipos para episódios de transtorno depressivo persistente, incluindo a depressão ansiosa, atípica, melancólica, catatônica, mista, psicótica, sazonal e periparto. Os quadros depressivos secundários ao uso de medicamentos e associados a patologias orgânicas tais como hipotireoidismo, apneia do sono, infarto agudo do miocárdio, acidente vascular cerebral, patologias crônicas, entre outras, podem ocorrer e devem ser afastados antes do diagnóstico de depressão maior ou persistente. Também é classificado o quadro de depressão menor quando não preenche todos os critérios de depressão maior, com pelo menos alteração no humor com um a três critérios da depressão com duração menor que duas semanas (Lyness, 2015).

A avaliação de fatores que influenciam a qualidade de vida dos estudantes de medicina assume papel relevante na atualidade do sistema de saúde brasileiro, em que se prioriza a formação de mais médicos com a criação de novas faculdades de Medicina e a ampliação do número de vagas. Há necessidade de se formar médicos tecnicamente competentes e capazes de atuar no sistema de saúde público e privado, onde o médico ocupa posição central para o adequado atendimento médico ambulatorial e hospitalar.

Este trabalho tem como propósito analisar o efeito do histórico de diagnóstico médico prévio de ansiedade ou depressão na qualidade de vida dos estudantes de medicina ainda no período acadêmico, utilizando o questionário WHOQOL-bref, desenvolvido pela OMS (The WHOQOL GROUP, 1998), e, assim, melhorar o desempenho acadêmico, contribuindo para a formação de um médico mais capaz, humano e centrado nas necessidades do paciente.

Avaliar marcadores que possam identificar grupos de estudantes com maior risco de ter sua qualidade de vida comprometida durante o curso médico poderá contribuir para o planejamento de ações para evitar prejuízos emocionais, acadêmicos e financeiros.

Os objetivos secundários deste artigo são: avaliar se há algum efeito na qualidade de vida quando o aluno de medicina refere praticar alguma religião e estudar a relação entre local de moradia dos familiares do acadêmico e histórico prévio de depressão, questões essas também mensuradas por instrumento específico.

2. PROCEDIMENTOS METODOLÓGICOS

2.1. Tipo de estudo

Esta pesquisa é caracterizada metodologicamente como observacional, populacional e transversal.

2.2. Amostra

A população foi constituída por acadêmicos do curso de Medicina da Universidade Nove de Julho (Uninove), São Paulo, Brasil, que ingressam semestralmente na Universidade. Como critério de inclusão, todos os alunos de medicina regularmente matriculados, de todos os semestres, foram convidados a participar da pesquisa. Os dados coletados foram compostos por amostras de conveniência, voluntária, com alunos que se encontravam em aula, nas salas de aula e nos campos de estágio, tendo sido obtida a participação de 405 alunos, do total de 600 acadêmicos em curso.

Todos os participantes receberam os esclarecimentos sobre o propósito da pesquisa e o termo de consentimento livre e esclarecido foi aplicado, somente após o qual os alunos que desejaram participar prosseguiram para a fase seguinte, com o autopreenchimento de questionário. Este estudo seguiu as orientações da Resolução 466/12 do Conselho Nacional de Saúde para experimentos com seres humanos, sendo aprovado pelo Comitê de Ética em Pesquisa com Seres Humanos da Uninove, de acordo com a declaração de Helsinki, sob protocolo número 014341.

2.3. Coleta de dados

A coleta dos dados ocorreu entre fevereiro e abril de 2014, por meio da aplicação do questionário da OMS para avaliação da qualidade de vida em seu formato resumido (WHOQOL-bref). Esse questionário tem com o objetivo avaliar o indivíduo em quatro domínios da qualidade de vida, sendo eles: capacidade física (sete questões), avaliação psicológica (seis questões), as relações sociais (três questões) e o meio ambiente em que o indivíduo se encontra (oito questões), totalizando 24 questões.

Além dos quatro domínios, o questionário apresenta mais duas perguntas gerais sobre qualidade de vida global. O preenchimento do WHOQOL-bref deve considerar as duas últimas semanas vividas pelos respondentes. O WHOQOL-bref é um resumo do QHOQOL-100 e incorporou o domínio nível de independência ao domínio físico e o domínio espiritualidade, religião e crença ao domínio psicológico.

As 24 facetas no WHOQOL-bref, avaliadas agora por uma pergunta e não mais por quatro perguntas, como no WHOQOL-100, são para o domínio físico: dor e desconforto, energia e fadiga, sono e repouso, mobilidade, atividades da vida cotidiana, dependência de medicação ou de tratamentos, capacidade de trabalho; para o domínio psicológico: sentimentos positivos, memória e concentração, autoestima, imagem corporal e aparência, sentimentos negativos e espiritualidade; para o domínio relações sociais: relações pessoais, suporte social, atividade sexual; domínio meio ambiente: segurança física e proteção, ambiente no lar, recursos financeiros, cuidados de saúde, oportunidades de acesso a novas informações e habilidades, recreação e lazer, ambiente físico, transporte.

As respostas obtidas em cada uma das perguntas foram classificadas em escala de 0 (zero) a 5 (cinco) com intervalo único, segundo o protocolo da OMS, sendo revertida a pontuação de três perguntas com sentido negativo (dor e desconforto, dependência de medicação ou de tratamentos e sentimentos negativos). Esse questionário foi traduzido e validado em nosso meio, podendo ser aplicado com segurança metodológica (Fleck et al., 2000). Os questionários que não foram totalmente preenchidos foram excluídos dessa análise.

Os resultados foram aplicados na equação sugerida pela OMS para obter o escore de cada domínio e seus respectivos escores finais, que variam em escala de 4 a 20 (média das facetas de cada domínio multiplicado por 4), e, depois, transformados na escala de 0 a 100 (subtrair 4 de cada domínio obtido na escala de 0 a 20 e multiplicar por 100 dividido por 16) (World Health Organization, 2012).

Além do WHOQOL-bref, foi anexado mais um questionário, em que o aluno deveria preencher os dados que caracterizavam o participante (ano na faculdade de medicina, sexo e idade). Foi perguntado se o aluno já teve diagnóstico médico prévio de ansiedade, pânico ou depressão, se pratica alguma religião e se sua família reside na cidade de São Paulo ou fora dela. Esse questionário foi aplicado a todos os 405 alunos simultaneamente ao WHOQOL-bref.

2.4. Tratamento estatístico

Inicialmente, os dados foram processados e coletados pelo programa Google Documents. Os dados estatísticos foram apresentados em médias e desvios padrões e foi utilizado o teste t de Student para amostras independentes, com a finalidade de comparar os resultados obtidos entre os grupos de acadêmicos que tinham histórico prévio de ansiedade, síndrome do pânico ou depressão dos que não tinham. Foram avaliados os seus respectivos domínios, sua média e desvios padrões. A mesma metodologia foi realizada para as perguntas referentes

à prática de alguma religião e se familiares do acadêmico residem em cidade outra que não São Paulo. Também foram avaliados os seus respectivos domínios e sua média, desvio padrões e o teste t de Student aplicado.

Também se comparou se há relação entre o histórico de ansiedade, pânico ou depressão e a prática de religião e a eventual residência dos pais na cidade de São Paulo. Foi utilizado o teste chi-quadrado com um grau de liberdade e o teste exato de Fisher. Para todos os cálculos estatísticos foi utilizado o STATA versão 13.

3. RESULTADOS

Foram avaliados 405 alunos de medicina da Universidade Nove de Julho, sendo que, destes, 177 (43,70%) são do sexo masculino e 228 (56,30%) do sexo feminino, distribuídos entre os seis anos de graduação da seguinte forma: 86 (21,23%) acadêmicos se encontravam no primeiro ano, 102 (25,19%) no segundo, 97 (23,95%) no terceiro, 59 (14,57%) no quarto, 35 (8,64%) no quinto e 26 (6,42%) no sexto. A média de idade foi de 23,55 anos com desvio padrão de 3,98. As características dos alunos estão resumidas nas tabelas 1 e 2.

Tabela 1 – Características dos estudantes de medicina quanto ao sexo e idade e cálculos de estatística descritiva média, desvio padrão, valor mínimo e valor máximo.

Variável	N (%)	Média	Desvio Padrão	Valor Mínimo	Valor Máximo
Idade	405 (100)	23,55 anos	3,98	18 anos	40 anos

Tabela 2 – Distribuição dos alunos de medicina estudados por ano letivo e percentual calculado.

Variável	N (%)
Ano acadêmico	405(100)
Primeiro	86 (21,23)
Segundo	102 (25,19)
Terceiro	97 (23,95)
Quarto	59 (14,57)
Quinto	35 (8,64)
Sexto	26 (6,42)

Do total de participantes deste trabalho, 140 (34,57%) tinham histórico de ansiedade, pânico ou depressão diagnosticados por médico. Esses estudantes de medicina apresentaram pior escore no domínio físico (57,27 vs 67,05 p<0,0010), no domínio psicológico (59,04 vs 68,31 p<0,0010), no domínio das relações sociais (67,14 vs 72,98 p =0,0044) e no domínio ambiental (55,78 vs 60,70 p=0,0018) em relação ao grupo que não tinha histórico de diagnóstico médico de ansiedade, pânico ou depressão (Tabela 3).

Tabela 3 – Análise da qualidade de vida conforme resultados obtidos no questionário WHOQOL-bref nos domínios físico, psicológico, relações interpessoais e ambientais para as variáveis diagnóstico prévio médico de ansiedade/pânico/depressão e religião. Foi utilizado o teste t de Student.

Variável e impacto		N (%) = 405	DF	DP	DRS	DA
Diagnóstico prévio – ansiedade, pânico e depressão	Não	265 (65,47)	67,05 (p<0,001)	68,31 (p<0,0010)	72,98 (p=0,0044)	60,70 (p=0,0018)
	Sim	140 (34,57)	57,27	59,04	67,14	55,78
Religião	Não	90 (22,22)	62,14 (p= 0,2656)	61,25 (p=0,0068)	69,07 (p=0,3018)	56,35 (0,0604)
	Sim	318 (77,78)	64,1	66,21	71,5	59,76

DF – domínio físico; DP – domínio psicológico; DRS – domínio de relações interpessoais; DA – domínio ambiental; TE – teste estatístico utilizado.

Quando avaliado o efeito de prática religiosa qualquer e qualidade de vida, foi detectado que os 318 (77,78%) alunos que responderam ter alguma religião apresentaram maior escore no domínio psicológico, quando comparados àqueles que não praticam nenhuma religião, diferença estatisticamente significativa (66,21 vs 61,25 p=0,0068) (Tabela 3). Nos outros domínios analisados, não foi observada diferença estatisticamente significativa entre os grupos analisados.

Foram comparadas entre si as variáveis, diagnóstico médico prévio de ansiedade, pânico ou depressão e a prática de religião, não tendo sido observada diferença estatisticamente significativa entre elas, conforme demonstra a tabela 4.

Tabela 4 – Tabela demonstrando a relação entre o diagnóstico médico prévio de ansiedade/depressão e religião, com teste estatístico Pearson chi2 e teste exato de Fisher.

Diagnóstico prévio de ansiedade/depressão	Tem religião	Não tem religião	TOTAL
	SIM	NÃO	
SIM	107 (76,43%)	33 (23,57%)	140 (100,00%)
NÃO	208 (78,49%)	57 (21,51%)	265 (100,00%)
TOTAL	315 (77,78%)	90 (22,22%)	405 (100,00%)

Pearson chi2(1) = 0.2253 PR =0.635; Teste exato de Fisher = 0.706.

A seguir, foi comparada a variável diagnóstico prévio de depressão à eventualidade de a família residir na cidade de São Paulo. Do grupo de alunos com histórico de ansiedade, pânico ou depressão, 75% não têm a família residindo em São Paulo, enquanto 62,64% não apresentam histórico de depressão, diferença estatisticamente significante (p=0.014) (tabela 5).

.

Tabela 5 – Tabela demonstrando a relação entre diagnóstico médico prévio de ansiedade/pânico/depressão e se a família do estudante reside em São Paulo ou fora, com os devidos testes estatísticos calculados Pearson chi^2 e teste exato de Fisher

Diagnóstico prévio ansiedade/pânico/depressão	Família reside em São Paulo	Família não reside em São Paulo	TOTAL
	SIM	NÃO	
SIM	35 (25,00%)	105 (75,00%)	140 (100,00%)
NÃO	99 (37,36%)	166 (62,64%)	265 (100,00%)
TOTAL	134 (33,09%)	271 (66,91%)	405 (100,00%)

Pearson $chi^2(1)$ = 6.3195 PR = 0.012 Teste exato de Fisher = 0.014.

4. ANÁLISE

O presente trabalho avaliou a qualidade de vida dos estudantes de medicina da UNINOVE utilizando o questionário da OMS traduzido e validado em nosso meio (Fleck et al., 2000). Esse método foi escolhido por apresentar metodologia amplamente utilizada para avaliação da qualidade de vida e possibilitar avaliar o aluno em quatro dimensões: física, psicológica, relações sociais e meio ambiente (The WHOQOL GROUP, 1998; World Health Organization, 2004).

Neste trabalho, foi demonstrado que os acadêmicos de Medicina que apresentam histórico de ansiedade, pânico ou depressão diagnosticado por médico têm pior índice de qualidade de vida nos quatro domínios analisados, domínio físico, psicológico, relação social e ambiental, em relação ao grupo que não possui histórico. Esse indicador, de fácil aplicação, pode ser utilizado para identificar grupos de alunos que demandam acompanhamento especial por tutores ou psicólogos, incentivo à prática esportiva, participação em grupos de interação, incentivo ao lazer, música, religião, artes e mesmo

terapias alternativas no sentido de prevenir prejuízos na qualidade de vida durante o curso médico.

A prevalência de alunos com histórico de ansiedade, pânico ou depressão (34,57%) foi a esperada em alunos de medicina. Investigação longitudinal realizada com alunos holandeses em duas escolas médicas com participação de 951 alunos mediu, por meio de escalas (BSI para depressão e BSI para ansiedade), e se encontraram incidências de depressão de 20%, 17% de ansiedade e 25% de outros transtornos mentais. Outras publicações baseadas em estudantes de faculdades de medicinas brasileiras e mesmo investigações internacionais demostram resultados similares para transtornos de ansiedade e depressão, entre outros transtornos mentais (Dyrbye, Harper, Durning, & Moutier, 2011; Dyrbye et al., 2006).

O ponto forte deste artigo é demonstrar que uma pergunta simples pode conduzir rapidamente à identificação de grupos de risco com piores índices de qualidade de vida e, portanto, mais sujeitos a desenvolver transtornos mentais.

Quando se avaliou a influência da religião nos índices de qualidade de vida, observou-se que os índices de qualidade de vida no domínio psicológico foram superiores no grupo de alunos que pratica alguma religião e que constituiu a maioria dos alunos (77,78%). Em números absolutos, os resultados foram superiores em todos os domínios, no entanto, apenas no domínio psicológico o resultado foi estatisticamente significante.

O domínio psicológico engloba questões sobre sentimentos positivos, pensar, aprender, memória e concentração, autoestima, imagem corporal e aparência, sentimentos negativos. Assim, verificamos que alunos de Medicina que praticam alguma religião mostraram melhor qualidade de vida. No entanto, os resultados da literatura são controversos e indicam necessidade de mais investigações para esclarecer a questão da religião como fator protetor (Ramakrishnan, 2015; Safara & Bhatia, 2015).

A maioria dos alunos da faculdade de Medicina estudada, que se localiza na cidade de São Paulo, são oriundos de cidades outras que não São Paulo (66,91%). Trata-se de aspecto interessante, em razão da atratividade e de maior concentração de vagas de Medicina na cidade de São Paulo, aspecto que vem mudando nas últimas duas décadas, com tendência a ser menos importante, até pelo fato de novas escolas estarem sendo abertas em outras cidades diferentes da capital paulista (Rocha, 2011).

Os alunos cujas famílias residem fora de São Paulo apresentaram maior percentual de histórico de ansiedade, pânico ou depressão, o que pode ser parcialmente explicado pelo distanciamento de pessoas queridas, isolamento e necessidade de adaptação à vida em outro local com costumes, hábitos de vida, violência, poluição, custo de vida certamente diferentes dos do local de onde vieram.

Diversas pesquisas internacionais relatam que os acadêmicos de medicina mostram altos índices de ansiedade, depressão e síndrome de burnout, com índices de desistência de 5,7% associados a isolamento, absenteísmo, depressão e um conjunto de sinais e sintomas que podem funcionar como sinal de alerta para atuação de grupos de ajuda (Baldassin et al., 2013; Dyrbye et al., 2005; Maher et al., 2013; Najafipour, Najafipour, & Yaktatalab, 2012).

Portanto, o uso de ferramentas de avaliação da qualidade de vida desses estudantes, que possam identificar os fatores de risco e de proteção, são de valia com o intuito de preservar, e até mesmo melhorar, a qualidade de vida deles, evitando assim as potenciais consequências a curto e também a logo prazo advindas de doenças como ansiedade e depressão, além de prejuízos financeiros e menor rendimento escolar.

No momento em que o Brasil tem a necessidade de formar mais médicos com bom embasamento técnico e humanista, e que estejam preparados para assumir toda a responsabilidade e proporcionar os resultados que a sociedade espera de sua atuação como cidadãos e como profissionais médicos, a avaliação dos alunos de Medicina, não somente dos ingressantes, mas ao longo do curso médico, se faz justificada. A Associação Médica Canadense possui uma política divulgada com o intuito de garantir bem-estar e melhorar os índices de depressão nos estudantes de Medicina (CMA Canadian Medical Association, 1998; Dyrbye et al., 2005; Puddester, 2001; Sullivan & Buske, 1998).

Os estudantes de Medicina podem desenvolver estratégias para lidar com a ansiedade e o estresse para se manterem saudáveis e maximizar suas habilidades para cuidar dos pacientes. Políticas de bem-estar e chamar a atenção das instituições, como o Conselho Federal de Medicina, órgãos regulamentadores da educação médica, incluindo o Ministério da Educação e o Ministério da Saúde, Associação Médica Brasileira, participação e mobilização da sociedade em geral, estudantes de medicina, médicos, prestadores de serviços de saúde e dirigentes de escolas médicas, no sentido de estruturar programas compatíveis com as políticas criadas, que resultem em melhorias no bem-estar dos estudantes de Medicina, médicos residentes e recém-formados e médicos em geral.

Os efeitos benéficos de atividades e de práticas relacionadas ao bem-estar dos alunos de medicina já são realidade em instituições internacionais e, futuramente, talvez sejam exigência dos órgãos regulamentadores do ensino médico. Essa medida auxiliaria na melhoria da qualidade de vida, geraria menos sofrimento e acarretaria aumento no desempenho acadêmico, além de contribuir para a

redução das taxas de depressão, ansiedade, entre outros transtornos mentais, melhora na capacidade física, cognitiva e psicológica.

Espera-se interferência positiva com impacto na resiliência do estudante, que estaria mais forte e seguro para cuidar de pessoas, tendo sua energia focada para realizar um bom diagnóstico, empatia e melhor satisfação do paciente e, com isso, aprimoramento da qualidade dos serviços de saúde.

Mais pesquisas serão realizadas, certamente, e devem ser implantados programas especiais que possam agir efetivamente na qualidade de vida do estudante de Medicina e de outros profissionais do setor, uma vez que o isolamento, estresse, competição, necessidade de obter resultados e pouca chance para o erro são algumas das principais causas do adoecimento dos profissionais da saúde (Dyrbye et al., 2005).

Estudantes de medicina com maior resiliência e capacidade de superar adversidades diante dos fatores estressantes e de desenvolver atitudes positivas frente às dificuldades e necessidades impostas pelo curso médico têm melhores índices de qualidade de vida e melhor percepção do ambiente educacional. Assim, a identificação de fatores de risco para o desenvolvimento de transtornos de ansiedade e depressivos é fundamental e será vista como pré-requisito para o bom funcionamento de uma escola de Medicina (Tempski et al., 2015).

Trabalhos recentes têm evidenciado que existem fatores que podem melhorar a condição de vida do estudante de Medicina, incluindo programas especiais já implantados em algumas faculdades, programas de melhoria da resiliência e estímulo à prática de atividade física, o que gera aumento da qualidade de vida e diminui os riscos de aparecimento de transtornos mentais, incluindo a melhoria do estresse, ansiedade, diminuição de incidência de episódios depressivos e reduz a incidência da síndrome de burnout (Bize, Johnson, & Plotnikoff, 2007; Pucci et al., 2012).

Há relato da implantação, com sucesso, de técnicas de relaxamento e massagens, melhora da autoestima e formas de lidar com estresse que parecem estar relacionadas à melhora no bem-estar do estudante de Medicina (Ranjbarkohn & Sajadinejad, 2010; Rong, Glozier, & Luscombe, 2011; Slonim, Kienhuis, Di Benedetto, & Reece, 2015; Velayudhan & Gayatridevi, 2010).

Uma limitação da análise realizada é ter sido um estudo transversal e, portanto, não se pode garantir que aqueles alunos de Medicina que tiveram ansiedade ou depressão não possam ter desenvolvido aqueles episódios antes ou no decorrer do curso médico. No entanto, a pergunta pode ser utilizada para se identificar grupos de estudantes com maior probabilidade de ter pior índice de qualidade de vida e, portanto, sujeitos a novos episódios de transtornos mentais, inclusive com risco de agravamento e evolução para quadros mais graves se não prontamente identificados e tratados.

5. CONSIDERAÇÕES FINAIS

Em conclusão, o histórico de ansiedade, pânico ou depressão diagnosticados pelo médico tem impacto negativo nos índices de qualidade de vida dos acadêmicos de Medicina quando avaliados por meio do questionário WHOQOL-bref, podendo a religião ser considerada fator protetor e relacionado à melhor qualidade de vida no aspecto psicológico do aluno de Medicina. Alunos que têm familiares que residem fora da cidade de São Paulo mostraram maior incidência de histórico de ansiedade, pânico ou depressão. Os indicadores encontrados neste estudo podem ser utilizados para identificar grupos de alunos mais expostos a uma pior qualidade de vida e a transtornos mentais comuns nos alunos de Medicina, até mesmo pela grande intensidade de energia, foco e regularidade de performance exigida nos seis anos de duração, em período integral, mais os períodos de plantões noturnos com privação de sono, distanciamento dos familiares e amigos e grande carga emocional exigida dos pacientes que procuram auxílio para a resolução de sua doença. Quem cuida da saúde dos outros precisa ter boa saúde e entender que a prática médica é prestação de serviço, em que o paciente procura qualidade, confiança, empatia e profissionais bem preparados e adequadamente treinados para a resolução de seu problema de saúde.

É importante ressaltar que são necessárias novas investigações para a identificação dos fatores que determinam vulnerabilidades e fatores de proteção, para que sejam trabalhados em um programa sério que avalie o aluno constantemente durante toda a sua formação médica, sem ser preconceituoso, persecutório e provocar mais angústias.

A pergunta sobre histórico de ansiedade, pânico ou depressão pode ser utilizada de imediato como indicador, bem como o incentivo à prática religiosa voluntária. Programas especiais para alunos provenientes de outras regiões fora de São Paulo entre os alunos de graduação em Medicina justificam-se, uma vez que foi aqui demonstrado seu efeito benéfico em vários aspectos avaliáveis da qualidade de vida.

Também pode-se inferir que a detecção precoce de estudantes com maior vulnerabilidade ao intenso ritmo da escola médica e a inclusão em programas especiais de melhoria de qualidade de vida possam contribuir para reduzir o estresse, a ansiedade, auxiliando na formação e manutenção de

laços afetivos entre familiares, amigos e, consequentemente, promover maior inserção social e na comunidade. Mais pesquisas para avaliar e entender melhor os fatores de risco e fatores de proteção, bem como programas especiais que aumentem a resiliência e bem-estar do estudante de medicina, podem ser implantados, e seus resultados, mensurados e analisados de modo a mitigar os efeitos do curso médico na qualidade de vida dos acadêmicos de medicina.

REFERÊNCIAS

Alonso, J., Lépine, J.-P., & ESEMeD/MHEDEA 2000 Scientific Committee. (2007). Overview of key data from the European Study of the Epidemiology of Mental Disorders (ESEMeD). *The Journal of Clinical Psychiatry*, *68 Suppl 2*, 3-9.

Ameringen, M. (2015). Comorbid anxiety and depression: Epidemiology, clinical manifestations, and diagnosis. In *UpToDate*. Waltham, MA: Denise S. Basow. Retrieved from <http://www.uptodate.com/contents/comorbid-anxiety-and-depression-epidemiology-clinical-manifestations-and-diagnosis?source=search_result&search=anxiety+disorders&selectedTitle=8~150>.

Andersson, N. W., Gustafsson, L. N., Okkels, N., Taha, F., Cole, S. W., Munk-Jørgensen, P., & Goodwin, R. D. (2015). Depression and the risk of autoimmune disease: a nationally representative, prospective longitudinal study. *Psychological Medicine*, 1-11. Retrieved from <http://doi.org/10.1017/S0033291715001488>.

Andrade, L., Caraveo-Anduaga, J. J., Berglund, P., Bijl, R. V., Graaf, R. D., Vollebergh, W., … Kessler, R. C. (2003). The epidemiology of major depressive episodes: results from the International Consortium of Psychiatric Epidemiology (ICPE) Surveys. *International Journal of Methods in Psychiatric Research*, *12*(1), 3-21.

Araújo, D., Gomes de Miranda, M. C., & Brasil, S. L. (2014). Formação de profissionais de saúde na perspectiva da integralidade. *Revista Baiana de Saúde Pública*, *31*, 20.

Association, A. P. (2013). *Diagnostic and Statistical Manual of Mental Disorders (DSM-5®)*. American Psychiatric Pub.

Baldassin, S., Silva, N., Toledo Ferraz Alves, T. C. de, Castaldelli-Maia, J. M., Bhugra, D., Nogueira-Martins, M. C. F., … Nogueira-Martins, L. A. (2013). Depression in medical students: cluster symptoms and management. *Journal of Affective Disorders*, *150*(1), 110-114. Retrieved from <http://doi.org/10.1016/j.jad.2012.11.050>.

Bize, R., Johnson, J. A., & Plotnikoff, R. C. (2007). Physical activity level and health-related quality of life in the general adult population: a systematic review. *Preventive Medicine*, *45*(6), 401-415.

Brazeau, C. M. L. R., Schroeder, R., Rovi, S., & Boyd, L. (2010). Relationships Between Medical Student Burnout, Empathy, and Professionalism Climate. *Academic Medicine*, *85*(10), S33-S36.

Brazeau, C. M. L. R., Shanafelt, T., Durning, S. J., Massie, F. S., Eacker, A., Moutier, C., … Dyrbye, L. N. (2014). Distress among matriculating medical students relative to the general population. *Academic Medicine: Journal of the Association of American Medical Colleges*, *89*(11), 1520-1525. Retrieved from <http://doi.org/10.1097/ACM.0000000000000482>.

Cataldo Neto, A., Cavalet, D., Bruxel, D. M., Kappes, D. S., & Silva, D. O. F. da S. (1998). O estudante de medicina e o estresse acadêmico. *Rev. Med. PUCRS*, *8*(1), 6-12.

Cavestro, J. M., & Rocha, F. L. (2006). Prevalência de depressão entre estudantes universitários. *J. Bras. Psiquiatr.*, *55*(4), 264-267.

Cerchiari, E. A. N., Caetano, D., & Faccenda, O. (2005). Prevalência de transtornos mentais menores em estudantes universitários. *Estudos de Psicologia*, *10*(3), 413-420.

Cheng, D., Poon, F., Nguyen, T., & Woodman, R. (2013). Stigma and perception of psychological distress and depression in Australian-trained medical students: Results from an inter-state medical school survey. *Psychiatry Research* (Query date: 2015-09-13). Retrieved from

<http://www.sciencedirect.com/science/article/pii/S0165178113001339>.

CMA Canadian Medical Association (Ed.). (1998). Physician health and well-being. *CMAJ : Canadian Medical Association Journal = Journal de l'Association Medicale Canadienne*, *158*(9), 1191-1200.

Conselho Federal de Medicna. (2007). *A saúde dos médicos no Brasil* (1st ed., Vol. 1). Brasília: Conselho Federal de Medicina. Retrieved from <http://www.portalmedico.org.br/include/asaudedosmedicosdobrasil.pdf>.

Cyranowski, J. M., Schott, L. L., Kravitz, H. M., Brown, C., Thurston, R. C., Joffe, H., … Bromberger, J. T. (2012). Psychosocial features associated with lifetime comorbidity of major depression and anxiety disorders among a community sample of mid-life women: the SWAN mental health study. *Depression and Anxiety*, *29*(12), 1050-1057. Retrieved from <http://doi.org/10.1002/da.21990>.

De Marco, O., Rossi, E., & Millan, L. (1999). *Considerações acerca do "erro médico" e de suas implicações psicológicas*. Casa do Psicólogo, 143-148.

Devi, S. (2011). Doctors in distress. *The Lancet*, (Query date: 2015-09-13). Retrieved from <http://www.thelancet.com/journals/lancet/article/PIIS0140-6736(11)60145-1/fulltext?rss=yes&utm_source=feedblitz&utm_medium=FeedBlitzEmail&utm_content=196607&utm_campaign=0>.

Donohue, J. M., & Pincus, H. A. (2007). Reducing the societal burden of depression: a review of economic costs, quality of care and effects of treatment. *PharmacoEconomics*, *25*(1), 7-24.

Downs, N., Feng, W., Kirby, B., McGuire, T., Moutier, C., & ... (2014). Listening to depression and suicide risk in medical students: the Healer Education Assessment and Referral (HEAR) program. *Academic ...* (Query date: 2015-09-13). Retrieved from <http://link.springer.com/article/10.1007/s40596-014-0115-x>.

Dyrbye, L. N., Harper, W., Durning, S., & Moutier, C. (2011). Patterns of distress in US medical students. *Medical Teacher* (Query date: 2015-09-13). Retrieved from <http://www.tandfonline.com/doi/abs/10.3109/0142159X.2010.531158>.

Dyrbye, L. N., Thomas, M. R., & Shanafelt, T. D. (2005). Medical student distress: causes, consequences, and proposed solutions. In *Mayo Clinic Proceedings* (Vol. 80, pp. 1613-1622). Elsevier. Retrieved from <http://www.sciencedirect.com/science/article/pii/S0025619611610574>.

Dyrbye, L. N., Thomas, M. R., & Shanafelt, T. D. (2006). Systematic review of depression, anxiety, and other indicators of psychological distress among US and Canadian medical students. *Academic Medicine*, *81*(4), 354-373.

Fleck, M., Louzada, S., Xavier, M., Chachamovich, E., Vieira, G., & Santos, L. (2000). Aplicação da versão em português do instrumento abreviado de avaliação da qualidade de vida. *Aplicação da versão em português do instrumento abreviado de avaliação da qualidade de vida "WHOQOL-Bref"*, *34*(2), 178-83.

Gabilondo, A., Rojas-Farreras, S., Vilagut, G., Haro, J., Fernandez, A., & Pinto-Meza, A. (2010). Epidemiology of major depressive episodes in a southern European country: results from the ESEMeD-Spain project. *J. Affect Disord.*, *120*(76).

Gold, J. A., Johnson, B., Leydon, G., Rohrbaugh, R. M., & Wilkins, K. M. (2015). Mental health self-care in medical students: a comprehensive look at help-seeking. *Academic Psychiatry: The Journal of the American Association of Directors of Psychiatric Residency Training and the Association for Academic Psychiatry*, *39*(1), 37-46. Retrieved from <http://doi.org/10.1007/s40596-014-0202-z>.

Goldman, M. L., Shah, R. N., & Bernstein, C. A. (2015). Depression and suicide among physician trainees: recommendations for a national response. *JAMA Psychiatry*, *72*(5), 411-412. Retrieved from <http://doi.org/10.1001/jamapsychiatry.2014.3050>.

Guerrero López, J. B., Heinze Martin, G., Ortiz de León, S., Cortés Morelos, J., Barragán Pérez, V., & Flores-Ramos, M. (2013). Factors that predict depression in medical students. *Gaceta Médica De México*, *149*(6), 598-604.

Hojat, M., Robeson, M., Damjanov, I., Veloski, J. J., Glaser, K., & Gonnella, J. S. (1993). Students' psychosocial characteristics as predictors of academic performance in medical school. *Academic Medicine: Journal of the Association of American Medical Colleges*, *68*(8), 635-637.

Honney, K., Buszewicz, M., Coppola, W., & Griffin, M. (2010). Students: Comparison of levels of depression in medical and non-medical students. *The Clinical Teacher*, *7*(3), 180-184.

Kessler, R. C., Berglund, P., Demler, O., Jin, R., Merikangas, K. R., & Walters, E. E. (2005). Lifetime prevalence and age-of-onset distributions of DSM-IV disorders in the National Comorbidity Survey Replication. *Archives of General Psychiatry*, *62*(6), 593-602. Retrieved from <http://doi.org/10.1001/archpsyc.62.6.593>.

Kessler, R. C., Ormel, J., Petukhova, M., McLaughlin, K. A., Green, J. G., Russo, L. J., … Alonso, J. (2011). Development of lifetime comorbidity in the World Health Organization world mental health surveys. *Archives of General Psychiatry*, *68*(1), 90-100.

Kim, B., & Roh, H. (2014). Depressive symptoms in medical students: prevalence and related factors. *Korean Journal of Medical Education*, *26*(1), 53-58. Retrieved from <http://doi.org/10.3946/kjme.2014.26.1.53>.

Krishnan, R. (2015). Unipolar depression in adults: Epidemiology, pathogenesis, and neurobiology. In *UpToDate*. Waltham, MA: Denise S. Basow. Retrieved from <http://www.uptodate.com/contents/unipolar-depression-in-adults-epidemiology-pathogenesis-and-neurobiology?source=search_result&search=unipolar+depression+in+adults&selectedTitle=3~150>.

Kumar, G., Jain, A., & Hegde, S. (2012). Prevalence of depression and its associated factors using Beck Depression Inventory among students of a medical college in Karnataka. *Indian Journal of Psychiatry* (Query date: 2015-09-12).

Lamers, F., Van Oppen, P., Comijs, H. C., Smit, J. H., Spinhoven, P., Van Balkom, A. J. L. M., … Penninx, B. W. J. H. (2011). Comorbidity patterns of anxiety and depressive disorders in a large cohort study: the Netherlands Study of Depression and Anxiety (NESDA). *The Journal of Clinical Psychiatry*, *72*(3), 341-348. Retrieved from <http://doi.org/10.4088/JCP.10m06176blu>.

Ludwig, A., Burton, W., Weingarten, J., Milan, F., Myers, D., & Kligler, B. (2015). Depression and stress amongst undergraduate medical students. *BMC Medical Education*, *15*(1), 141.

Lyness, J. M. (2015). Unipolar depression in Adults: Assessment and diagnosis. In *UpToDate*. Waltham, MA: Denise S. Basow. Retrieved from <http://www.uptodate.com/contents/unipolar-depression-in-adults-assessment-and-diagnosis?source=search_result&search=unipolar+depression+in+adults&selectedTitle=2~150>.

Magee, W., Eaton, W., Wittchen, H., McGonagle, K., & Kessler, R. (1996). Agoraphobia, simple phobia, and social phobia in the national comorbidity survey. *Arch Gen Psychiatry*, *53*(159).

Maher, B. M., Hynes, H., Sweeney, C., Khashan, A. S., O'Rourke, M., Doran, K., … Flynn, S. O. (2013). Medical school attrition-beyond the statistics a ten year retrospective study. *BMC Medical Education*, *13*, 13. Retrieved from <http://doi.org/10.1186/1472-6920-13-13>.

Martin, J. (2010). Stigma and student mental health in higher education. *Higher Education Research & Development* (Query date: 2015-09-12). Retrieved from <http://www.tandfonline.com/doi/abs/10.1080/07294360903470969>.

Mosley, T. H. J., Perrin, S. G., Neral, S. M., Dubbert, P. M., Grothues, C. A., & Pinto, B. M. (1994). Stress, coping, and well-being among third-year medical students. *Academic Medicine*, *69*(9), 765-7.

Najafipour, S., Najafipour, F., & Yaktatalab, S. (2012). P-509-The prevalence depression and relationships with academic failure on students of Jahrom University of Medical Science. *European Psychiatry* (Query date: 2015-09-13). Retrieved from <http://www.sciencedirect.com/science/article/pii/S092493381274676X>.

Nogueira, M. I. (2009). As mudanças na educação médica brasileira em perspectiva: reflexões sobre a emergência de um novo estilo de pensamento. *Rev. Bras. Educ. Med.*, 262-270.

Paula, J. dos A. de, Borges, A. M. F. S., Bezerra, L. R. A., Parente, H. V., Paula, R. C. dos A. de, Wajnsztejn, R., … Abreu, L. C. de. (2014). Prevalence and factors associated with depression in medical students. *Revista Brasileira de Crescimento e Desenvolvimento Humano*, *24*, 274-281.

Phelan, S. M., Burgess, D. J., Puhl, R., Dyrbye, L. N., Dovidio, J. F., Yeazel, M., … Van Ryn, M. (2015). The Adverse Effect of Weight Stigma on the Well-Being of Medical Students with Overweight or Obesity: Findings from a National Survey. *Journal of General Internal Medicine*, *30*(9), 1251-1258. Retrieved from <http://doi.org/10.1007/s11606-015-3266-x>.

Pucci, G. C. M. F., Rech, C. R., Fermino, R. C., & Reis, R. S. (2012). Associação entre atividade física e qualidade de vida em adultos. *Revista de Saúde Pública*, *46*, 166-179.

Puddester, D. (2001). The Canadian Medical Association's Policy on Physician Health and Well-being. *Western Journal of Medicine*, *174*(1), 5-7.

Quince, T., Wood, D., Parker, R., & Benson, J. (2012). Prevalence and persistence of depression among undergraduate medical students: a longitudinal study at one UK medical school. *BMJ Open* (Query date: 2015-09-13). Retrieved from <http://bmjopen.bmj.com/content/2/4/e001519.short>.

Ramakrishnan, P. (2015). "You are here": locating "spirituality" on the map of the current medical world. *Current Opinion in Psychiatry*, *28*(5), 393-401.

Ranjbarkohn, Z., & Sajadinejad, M. (2010). Effect of assertiveness training on self-esteem and depression in students of Isfahan University of Medical Sciences. *Journal of Birjand University of Medical Sciences* (Query date: 2015-09-13). Retrieved from <http://journal.bums.ac.ir/browse.php?a_code=A-10-1-382&slc_lang=en&sid=1&sw=ER>.

Roberts, L. W., Warner, T. D., Lyketsos, C., Frank, E., Ganzini, L., & Carter, D. (2001). Perceptions of academic vulnerability associated with personal illness: a study of 1,027 students at nine medical schools. Collaborative Research Group on Medical Student Health. *Comprehensive Psychiatry*, *42*(1), 1-15.

Rocha, J. H. (2011). O Ensino Médico e a distribuição dos Médicos. In *Saúde Solidária – seguridade médica para todos*. Retrieved from <http://www.escolasmedicas.com.br/art_det.php?cod=214>.

Roh, M., Jeon, H., Kim, H., Han, S., & Hahm, B. (2010). The prevalence and impact of depression among medical students: a nationwide cross-sectional study in South Korea. *Academic Medicine* (Query date: 2015-09-13). Retrieved from <http://journals.lww.com/academicmedicine/Abstract/2010/08000/The_Prevalence_and_Impact_of_Depression_Among.28.aspx>.

Rong, Y., Glozier, N., & Luscombe, G. (2011). Improving knowledge and attitudes towards depression: a controlled trial among Chinese medical students. *BMC* ... (Query date: 2015-09-13).

Rossi, E., De Marco, O. L., & Millan, L. (1991). Reflexões sobre o suicídio entre os estudantes de medicina. *Rev. Med. (São Paulo)*, *70*(1/2), 28-30.

Rubin, R. (2014). Recent suicides highlight need to address depression in medical students and residents. *JAMA*, *312*(17), 1725-1727. Retrieved from <http://doi.org/10.1001/jama.2014.13505>.

Safara, M., & Bhatia, M. (2015). Relationship of Religious Beliefs with Anxiety and Depression. *Medind.nic.in* (Query date: 2015-09-13).

Sansone, R. A., & Sansone, L. A. (2009). Physician Suicide: A Fleeting Moment of Despair. *Psychiatry (Edgmont)*, *6*(1), 18-22.

Schwenk, T., Davis, L., & Wimsatt, L. (2010). Depression, stigma, and suicidal ideation in medical students. *JAMA* (Query date: 2015-09-13).

Sharac, J., Mccrone, P., & Clement, S. (2010). The economic impact of mental health stigma and discrimination: a systematic review. *Epidemiologia e Psichiatria Sociale* (Query date: 2015-09-13). Retrieved from <http://journals.cambridge.org/abstract_S1121189X00001159>.

Slonim, J., Kienhuis, M., Di Benedetto, M., & Reece, J. (2015). The relationships among self-care, dispositional mindfulness, and psychological distress in medical students. *Medical Education Online*, *20*, 27924.

Sullivan, P., & Buske, L. (1998). Results from CMA's huge 1998 physician survey point to a dispirited profession. *CMAJ: Canadian Medical Association Journal = Journal de l'Association Medicale Canadienne*, *159*(5), 525-528.

Tan, S. T., Sherina, M. S., Rampal, L., & Normala, I. (2015). Prevalence and predictors of suicidality among medical students in a public university. *The Medical Journal of Malaysia*, *70*(1), 1-5.

Tempski, P., Santos, I. S., Mayer, F. B., Enns, S. C., Perotta, B., Paro, H. B. M. S., … Martins, M. A. (2015). Relationship among Medical Student Resilience, Educational Environment and Quality of Life. *PloS One*, *10*(6), e0131535. Retrieved from <http://doi.org/10.1371/journal.pone.0131535>.

The WHOQOL GROUP (Ed.). (1998). Development of the World Health Organization WHOQOL-BREF quality of life assessment. *Development of the World Health Organization WHOQOL-BREF Quality of Life Assessment. The WHOQOL Group*, *28*(3), 551-8.

Vankar, J., & Prabhakaran, A. (2014). Depression and stigma in medical students at a private medical college. *Indian Journal of Pshycol Med* (Query date: 2015-09-13).

Velayudhan, A., & Gayatridevi, S. (2010). Efficacy of behavioral intervention in reducing anxiety and depression among medical students. *Industrial Psychiatry Journal* (Query date: 2015-09-13).

Villanueva, T., & Haivas, I. (2006). Studying medicine and quality of life, *14*, 136-176.

Wallace, J. (2010). Mental health and stigma in the medical profession. *Public, Environmental & Occupational Health* (Query date: 2015-09-13). Retrieved from <http://hea.sagepub.com/content/early/2010/12/21/1363459310371080.abstract>.

Wimsatt, L., Schwenk, T., & Sen, A. (2015). Predictors of depression stigma in medical students: potential targets for prevention and education. *American Journal of Preventive Medicine* (Query date: 2015-09-13). Retrieved from <http://www.sciencedirect.com/science/article/pii/S0749379715001439>.

World Health Organization (Ed.). (2012). WHOQOL User Manual, Division of Mental Health and Prevention of Substance Abuse, World Health Organization. Retrieved from <http://www.who.int/iris/handle/10665/77773>.

Zoccolillo, M., Murphy, G. E., & Wetzel, R. D. (1986). Depression among medical students. *Journal of Affective Disorders*, 11(1), 91-96. Retrieved from <http://doi.org/10.1016/0165-0327(86)90065-0>.
