# Supplementary material for: A cross-sectional study of sociodemographic factors and their influence on quality of life in medical students at Sao Paulo, Brazil
Source: PLoS One. 2017 Jul 10;12(7):e0180009. doi: 10.1371/journal.pone.0180009 (PMC5503183; doi:10.1371/journal.pone.0180009)
Supplement: S3 File — (PDF) [file pone.0180009.s003.pdf]

ConScientiae Saúde
ISSN: 1677-1028
conscientiaesaude@uninove.br
Universidade Nove de Julho
Brasil

Ivo Serinolli, Mario; El-Mafarjeh, Elias

Impacto da prática de atividade física na qualidade de vida dos acadêmicos de Medicina da Universidade Nove de Julho (Uninove)

ConScientiae Saúde, vol. 14, núm. 4, 2015, pp. 627-633
Universidade Nove de Julho
São Paulo, Brasil

Disponível em: http://www.redalyc.org/articulo.oa?id=92945642014

Como citar este artigo
Número completo
Mais artigos
Home da revista no Redalyc

Sistema de Informação Científica
Rede de Revistas Científicas da América Latina, Caribe , Espanha e Portugal
Projeto acadêmico sem fins lucrativos desenvolvido no âmbito da iniciativa Acesso Aberto

DOI:10.5585/ConsSaude.v14n4.5958 Recebido em 4 set. 2015 / aprovado em 18 dez. 2015

Impacto da prática de atividade física na qualidade de vida dos acadêmicos de Medicina da Universidade Nove de Julho (Uninove)

Impact of physical activity practice on quality of life of Medical Students, Nove de Julho University (Uninove)

Mario Ivo Serinolli[1], Elias El-Mafarjeh[2]

[1] Professor Permanente do Mestrado Profissional Gestão em Sistemas de Saúde Universidade Nove de Julho – UNINOVE – São Paulo, SP - Brasil.
[2] Graduando em Medicina da Universidade Nove de Julho – UNINOVE – São Paulo, SP - Brasil.

Endereço de Correspondência:
Mario Ivo Serinolli
Avenida Francisco Matarazzo N. 612 Prédio C – 1° andar
CEP 05001-000 São Paulo – SP [Brasil]
mserinolli@gmail.com

Resumo

Introdução: A prática de atividade física é recomendada para a prevenção das doenças não transmissíveis e melhora da qualidade de vida. **Objetivo:** Este trabalho objetivou avaliar os impactos do exercício físico na vida dos estudantes de Medicina. **Método:** Foi realizado estudo transversal com aplicação do questionário da Organização Mundial da Saúde (WHOQOL-BREF) e perguntas relacionadas à atividade física em 405 alunos de medicina da cidade de São Paulo, Brasil. **Resultados:** Os alunos que praticam exercícios físicos em competições esportivas e os que o fazem regularmente por pelo menos trinta minutos igual ou mais que três vezes por semana obtiveram melhor índice de qualidade de vida demonstrado em quase todos os domínios ($p<0{,}005$). **Conclusão:** Na amostra analisada, acadêmicos de Medicina que praticam atividades físicas competitivas ou com alta frequência semanal têm melhor qualidade de vida quando comparados àqueles que o fazem infrequentemente e aos sedentários.

Descritores: Qualidade de Vida; Estudantes de Medicina; Estresse Psicológico; Relações Interpessoais; Atividade Física.

Abstract

Introduction: Regular physical activity is recommended for the prevention of non-communicable diseases and to improve quality of life. **Objective:** This study aims to evaluate the effects of physical activity in the quality of life of medical students. **Method:** We used a cross-sectional study with application of the World Health Organization questionnaire (WHOQOL-BREF) and questions related to physical activity in 405 medical students of Sao Paulo, Brazil. **Results:** Students who participate in sports competitions and those who do physical activities, regularly, for at least thirty minutes three times a week or more have better quality of life index demonstrated in almost all domains ($p <0.005$). **Conclusion:** In this study, medical students who are engaged in sports competitions and those who do physical activities, regularly, for at least thirty minutes three times a week have better quality of life when compared to those who do it infrequently or are sedentary.

Keywords: Quality of Life; Students, Medical; Psychological Stress; Interpersonal Relations; Physical Activity.

Introdução

O curso de Medicina é mundialmente conhecido pela sua complexidade e dificuldade, devido à grande exigência aos alunos como tempo prolongado de estudo, sacrifícios, isolamento social e resistência física e emocional. A relação entre o estresse e a Medicina é estudada em diversos países, principalmente seu impacto na qualidade de vida do estudante de graduação [1-11].

Comumente, encontramos relatos de distúrbios de ansiedade, consumo de drogas, depressão e até mesmo casos de suicídio em maior número em estudantes e profissionais médicos do que na população em geral – reflexo das condições a que os médicos e estudantes são submetidos em suas atividades. Há relatos de que a síndrome de *Burnout*, devido à exaustão emocional, desumanização e redução da realização profissional é mais observada nesse grupo de profissionais, decorrente da constante sensação de impotência gerada pelo contato constante com casos pacientes terminais e do contato constante com a morte, além da pressão constante sofrida no trabalho e no meio acadêmico, o que afeta diretamente a qualidade de vida do profissional [8-14].

Segundo pesquisa realizada pela *Canadian Medical Association*, que avaliou 3.520 profissionais de saúde e observou que as principais causas desse perfil insatisfatório são a elevada carga de trabalho, os impactos negativos na vida familiar e pessoal, a escolha da profissão e insatisfação com a área de escolha. No Brasil, recentemente, o Conselho Federal de Medicina publicou um artigo sobre a qualidade de vida do profissional no país em que foi apontado que 5% dos médicos se sentem sem esperança e apresentam pensamentos suicidas. Com isso, pode-se notar que as condições estressantes do trabalho para médicos e estudantes não é exclusividade do Brasil [5,6, 14,15].

Trabalhos recentes têm evidenciado que existe relação positiva entre a prática de atividade física e o aumento da qualidade de vida. Essa relação é conflitante em diferentes sociedades, entretanto, mesmo com essa relação conflitante, a associação positiva existe e varia de acordo com os domínios de qualidade de vida avaliados [16-21].

A atividade física, quando praticada de forma regular, além de levar ao aumento da qualidade de vida, pode impactar na vida dos profissionais médicos e em estudantes de medicina, uma vez que a prática libera hormônios como endorfinas que agem no sistema nervoso, reduzindo o impacto estressor do ambiente e, com isso, pode prevenir ou reduzir transtornos depressivos [17, 21,23]. Além disso, a prática de exercícios físicos contribui para a prevenção de doenças não transmissíveis, como doença cardíaca, diabetes tipo 2, câncer de mama e cólon, por exemplo [23,24].

O exercício físico promove melhora na capacidade respiratória, na reserva cardíaca, nos reflexos, na força muscular, na memória recente, na cognição e nas habilidades sociais, promovendo a melhora do funcionamento do corpo dos esportistas. Durante a realização de exercício físico, ocorre a liberação da b-endorfina e da dopamina pelo organismo, causando efeito tranquilizante e analgésico na prática regular, fazendo com que, em geral, o praticante tenha estado de equilíbrio psicossocial mais estável frente às ameaças do meio externo, o que, para os estudantes de medicina, é algo quase que vital para a boa prática e estudos [17, 23,25,26].

Outro ponto importante é que a prática constante de exercício físicos promove a diminuição da depressão e da ansiedade no atleta, o que é extremamente importante para os profissionais de saúde. O mecanismo para que isso o ocorra é o mesmo do relaxamento pós-treino anteriormente citado, a liberação da b-endorfina e da dopamina pelo organismo [17, 23].

Devido aos dados escassos correlacionando o impacto da prática de exercícios físicos na vida dos acadêmicos de Medicina, este trabalho tem como objetivo analisar como os efeitos da prática de exercícios físicos alteram a qualidade de vida dos estudantes ainda no período acadêmico, utilizando o questionário WHOQOL-BREF desenvolvido pela Organização Mundial de Saúde (OMS) [27,28].

Materiais e métodos

Tipo de estudo

Esta pesquisa é caracterizada metodologicamente como observacional, populacional e transversal.

Amostra

A população foi constituída por acadêmicos do curso de Medicina da Universidade Nove de Julho (Uninove), São Paulo, Brasil que conta com aproximadamente 600 alunos que ingressam semestralmente na Universidade. Como critério de inclusão, todos os alunos de medicina regularmente matriculados, de todos os semestres, foram convidados a participar do estudo. Os dados coletados foram compostos de forma randômica, voluntária, com alunos que se encontravam em aula, nas salas de aulas e nos campos de estágio, tendo sido obtida a participação de 405 alunos.

Todos os participantes deste estudo receberam os esclarecimentos sobre o propósito da pesquisa e o termo de consentimento livre e esclarecido foi aplicado, somente após o quê, os alunos que desejaram participar prosseguiram para a fase seguinte, com o autopreenchimento de questionário. Este estudo seguiu as orientações da Resolução 466/12 do Conselho Nacional de Saúde para experimentos com seres humanos, sendo aprovado pelo Comitê de Ética em Pesquisa com Seres Humanos da Uninove, de acordo com a declaração de Helsinki, sob protocolo número 014341.

Coleta de dados

A coleta dos dados ocorreu entre fevereiro e abril de 2014, por meio da aplicação do questionário da Organização Mundial da Saúde para avaliação da qualidade de vida no seu formato curto (WHOQOL-BREF). Esse questionário tem com o objetivo avaliar o indivíduo em quatro domínios da qualidade de vida, sendo eles: capacidade física (sete questões), avaliação psicológica (seis questões), as relações sociais (três questões) e o meio ambiente em que o indivíduo se encontra (oito questões), totalizando 24 questões. Além dos quatro domínios, o questionário apresenta mais duas perguntas gerais sobre qualidade de vida global. O preenchimento do WHOQOL-BREF deve considerar as duas últimas semanas vividas pelos respondentes [19].

As respostas foram classificadas em escala de 0 (zero) a 5 (cinco) com intervalo único, segundo o protocolo da OMS. Os resultados foram aplicados na equação sugerida pela OMS para obter o escore de cada domínio e seus respectivos escores finais que variam em escala de 4 a 20. Os questionários que não foram totalmente preenchidos foram excluídos desta análise.

Além do WHOQOL-BREF, foi anexado mais um questionário em que o aluno deveria preencher dados que caracterizavam o participante (ano na faculdade de medicina, sexo e idade) e dados em relação a avaliação física e esportiva do aluno (peso, altura e frequência de atividades física). Esse questionário foi aplicado a todos os 405 alunos simultaneamente ao WHOQOL-BREF.

Foram feitas duas perguntas sobre atividade física. Uma delas, se o aluno realiza alguma prática esportiva dentre aquelas oferecidas oficialmente pela Faculdade, com o objetivo de participar de competições esportivas, representando a Faculdade de Medicina. A outra questão formulada (independente) foi se o aluno exerce alguma prática esportiva dentro ou fora da faculdade por mais de 30 minutos até duas vezes por semana, três ou mais vezes por semana ou não pratica atividade física.

Tratamento estatístico

Inicialmente, os dados foram processados e coletados pelo programa Google Documents. Os dados estatísticos foram apresentados em médias e desvios padrões e foi utilizado o teste T de Student para amostras independentes, com a finalidade de comparar os resultados obtidos entre os grupos de acadêmicos que praticam atividades físicas e o grupo de acadêmicos que

não praticam atividades físicas, para participar de competições esportivas da Faculdade de Medicina. Quando avaliado o grupo de acadêmicos que praticam atividades físicas por mais de 30 minutos, ele foi subdivido em três grupos: os alunos que não praticam atividade física, os alunos que praticam atividades físicas até duas vezes por semana (dentro e fora da Faculdade) e alunos que praticam atividades físicas três ou mais vezes por semana (dentro e fora da faculdade) e foram avaliados os seus respectivos domínios e sua média, desvios padrões e ANOVA calculados com o teste de Bonferroni.

Resultados

Foram avaliados 405 alunos de medicina da Universidade Nove de Julho, sendo que, desses, 177 (43,70%) são do sexo masculino e 228 (56,30%) do sexo feminino, distribuídos entre os seis anos de graduação da seguinte forma: 86 (21,23%) acadêmicos se encontravam no primeiro ano, 102 (25,19%) no segundo, 97 (23,95%) no terceiro, 59 (14,57%) no quarto, 35 (8,64%) no quinto e 26(6,42%) no sexto. A média de idade foi de 23,55 anos com desvio padrão de 3,98. O peso e estatura dos alunos foram tabulados. As características dos alunos estão resumidas nas tabelas 1 e 2.

Os alunos que praticam exercícios físicos com a finalidade de participar de competições pela faculdade obtiveram melhor escore nos domínios físico, psicológico, relações sociais, e ambiental em relação ao grupo que não realiza atividade física oferecida pela Faculdade de Medicina (Tabela 3).

Os resultados para os alunos que praticam atividade física (pela faculdade, clubes, academias, parques) por pelo menos 30 minutos por até duas vezes na semana e os que praticam três ou mais vezes por semana foram superiores quando comparados aos que não praticavam exercícios físicos nos domínios físico, psicológico e ambiental (Tabela 3). O teste de Bonferroni foi estatisticamente significante nos domínios físico (p=0,012), psicológico (p=0,028) e ambiental (p=0,014) quando se comparou a prática de atividade física por pelo menos trinta minutos por três ou mais vezes por semana comparado aos que não possuem prática de exercícios físicos.

Tabela 2: Distribuição dos alunos por ano letivo na Faculdade de Medicina

Ano acadêmico	N (%) = 405
Primeiro	86 (21,23)
Segundo	102 (25,19)
Terceiro	97 (23,95)
Quarto	59 (14,57)
Quinto	35 (8,64)
Sexto	26 (6,42)

Tabela 1: Características dos estudantes de medicina estudados

Variável	N(%)	Média	Desvio Padrão	Valor Mínimo	Valor Máximo
idade					
Masculino	177 (43,70)	23,57	4,13	18 anos	40 anos
Feminino	228 (56,30)	23,54	3,87	18 anos	40 anos
Total	405 (100,00)	23,55	3,98	18 anos	40 anos
Peso					
Masculino	177 (43,70)	82,44	13,59	57Kg	139Kg
Feminino	228 (56,30)	60,76	11,22	43Kg	115Kg
Total	405 (100,00)	70,24	16,34	43Kg	139Kg
Altura					
Masculino	177 (43,70)	177,9	5,87	162cm	197cm
Feminino	228 (56,30)	164,2	6,3	150cm	179cm
Total	405 (100,00)	170,19	9,14	150cm	197cm

Tabela 3: Análise da qualidade de vida conforme resultados obtidos no questionário WHOQOL-BREF nos domínios físico, psicológico, relações interpessoais e ambiental

Variável e impacto	N (%) = 405	DF	DP	DRS	DA	TE
Atividade esportiva na faculdade						
Não	296 (73,09)	**62,69 (p=0,0279)**	**63,61 (p=0,0012)**	**69,17 (p=0,0024)**	**57,67 (p=0,0036)**	t
Sim	109 (26,91)	**66,31**	**69,18**	**75,84**	**62,61**	
Atividade física pelo menos 30 minutos						
Não	165 (40,78)	**61,71 (p=0,0145)**	**63,06 (p=0,034)**	69,75 (p=0,41)	**57,14 (p=0,0141)**	anova
Até duas vezes por semana	116 (28,64)	**63,18**	**65,16**	70,69	**58,19**	
Três ou mais vezes por semana	124 (30,62)	**66,73**	**67,81**	72,85	**62,25**	

DF- domínio físico; DP- domínio psicológico; DRS - domínio de relações interpessoais; DA - domínio ambiental; TE - teste estatístico utilizado.

Discussão

Neste trabalho, foi constado que os acadêmicos de Medicina que praticam exercícios físicos na Faculdade de Medicina e participam das competições esportivas obtiveram melhor domínio físico, psicológico, relação social e ambiental, em relação ao grupo que não realiza nenhum tipo de atividade física. A hipótese de que a prática de atividades esportivas teria impacto relevante na vida dos alunos foi confirmada.

Quando se avaliou a intensidade da prática esportiva, observou-se que os índices de qualidade de vida foram superiores nos domínios físico, psicológico e ambiental no grupo de alunos que praticam três ou mais vezes por semana por pelo menos 30 minutos. Curiosamente, embora o índice seja superior também no domínio relação social, ele não foi estatisticamente significante. No entanto, aqueles alunos que realizam prática esportiva na faculdade com a finalidade de participar de competições esportivas do setor, apresentaram índices superiores nos quatro domínios incluindo o de relação social.

Segundo Hassed *et al.* [29] e Alves *et al.* [30], existe diferença estatisticamente significante na qualidade de vida entre os alunos do primeiro e sexto ano da faculdade de Medicina. Porém, essa melhora foi encontrada apenas no domínio psicológico. Essa diferença entre os diferentes anos não foi analisada aqui, uma vez que o objetivo foi analisar o impacto da prática esportiva do aluno de medicina de forma geral, em todos os anos da faculdade.

Os efeitos benéficos da prática acadêmica como redução da depressão, ansiedade, melhora na capacidade respiratória, na reserva cardíaca, nos reflexos, na força muscular, na memória recente, na cognição e nas habilidades sócias são os possíveis fatores que devem influenciar a melhora da qualidade de vida desse grupo, uma vez que o isolamento, estresse e os fatores relacionados a síndrome de Burnout são das principais causas do adoecimento dos profissionais da saúde.

O presente estudo avaliou a qualidade de vida dos estudantes de medicina da UNINOVE utilizando o questionário da Organização Mundial de Saúde – WHOQOL – BREF. (The World Health Organization Quality of Life (WHOQOL)-BREF, World Health Organization 2004). Esse método foi escolhido por apresentar metodologia amplamente utilizada para avaliação da qualidade de vida e possibilitar avaliar o aluno em quatro dimensões: física, psicológica, relações sociais e meio ambiente [23, 24].

Conclusão

A prática de atividade física tem impacto positivo nos índices de qualidade de vida dos acadêmicos de Medicina, o que pôde ser constatado por meio do questionário WHOQOL-BREF e análise de seu perfil esportivo e intensidade da prática esportiva. Na amostra analisada, acadêmicos de medicina que praticam atividades físicas competitivas ou com alta frequência se-

manal têm melhor qualidade de vida quando comparados àqueles que o fazem infrequentemente ou sedentários.

Este estudo evidenciou que a participação dos estudantes em competições esportivas revela índice superior com significância estatística em todos os domínios estudados (físico, psicológico, relações sócias e ambiental). Para os alunos que praticam atividade física, quanto maior a intensidade, maior o índice de qualidade de vida demonstrada em três domínios (físico, psicológico e ambiental). Entretanto, são necessários novos estudos para a identificação dos fatores que determinam essas alterações recorrentes da prática de atividades física na qualidade de vida do estudante de Medicina.

As atividades físicas entre os alunos de graduação em Medicina devem ser estimuladas, uma vez que foi demonstrado aqui seu efeito benéfico em vários aspectos avaliáveis da qualidade de vida. Também, pode-se inferir que a prática de atividade física em estudantes de Medicina possa contribuir para reduzir o estresse, a ansiedade, auxiliando na formação e manutenção de laços afetivos entre familiares, amigos e, consequentemente, promover maior inserção social e na comunidade. Estudos devem ser realizados para avaliar a correlação da melhor qualidade de vida, prática esportiva e melhor performance acadêmica.

Referências

1. Ludwig A, Burton W, Weingarten J, Milan F, Myers D, Kligler B. Depression and stress amongst undergraduate medical students. BMC Med Educ. 2015; 15(1): 141-145.
2. Rizvi F, Qureshi A, Rajput A, Afzal M. Prevalence of depression, anxiety and stress (by DASS scoring system) among medical students in Islamabad, Pakistan. Br J Med Med Res. 2015; 8(1): 69–75.
3. Nacar M, Cetinkaya F, Baykan Z, Yilmazel G, Elmali F. Hazardous Health Behaviour among Medical Students: a Study from Turkey. Asian Pac J Cancer Prev APJCP. 2015; 16(17): 7675–81.
4. Lins L, Carvalho FM, Menezes MS, Porto-Silva L, Damasceno H. Health-related quality of life of students from a private medical school in Brazil. Int J Med Educ. 2015; 6: 149–54.
5. Cataldo Neto A, Cavalet D, Bruxel DM, Kappes DS, Silva DOF. O estudante de medicina e o estresse acadêmico. Revista Médica da PUCRS 1998; 8(1): 6-12.
6. DeMarco OLN, Rossi E, Millan LR. Considerações acerca do "erro médico" e de suas implicações psicológicas. In: Millan LR, De Marco OLN, Rossi E, Arruda PCV. O Universo psicológico do futuro médico, vocação, vicissitudes e perspectivas. São Paulo: Casa do Psicólogo, 1999. p. 143-148.
7. Villanueva T, Haivas I. Studying medicine and quality of life. Student BMJ 2006; 14: 133-76.
8. Eckleberry-Hunt J, Lick D. Physician Depression and Suicide: A Shared Responsibility. Teach Learn Med. 2015; 27(3): 341–5.
9. Paro HB, Silveira PS, Perotta B, Gannam S, Enns SC, Giaxa RR, et al. Empathy among medical students: is there a relation with quality of life and burnout? PLoS One. Apr. 4, 2014; 9(4): e94133–e94133.
10. Tan ST, Sherina MS, Rampal L, Normala I. Prevalence and predictors of suicidality among medical students in a public university. Med J Malaysia. Feb, 2015; 70(1): 1–5.
11. Dyrbye LN, Eacker A, Durning SJ, Brazeau C, Moutier C, Massie FS, et al. The Impact of Stigma and Personal Experiences on the Help-Seeking Behaviors of Medical Students With Burnout. Acad Med J Assoc Am Med Coll. Feb 3, 2015; 90(7): 961–9.
12. Wimsatt L, Schwenk T, Sen A. Predictors of depression stigma in medical students: potential targets for prevention and education. Am J Prev Med. 2015; 49(5): 703–14.
13. Costa EF de O, Santos SA, Santos ATR de A, Melo EV de, Andrade TM de. Burnout Syndrome and Associated Factors Among Medical Students: A Cross-Sectional Study. Clinics. 2012; 67: 573–80.
14. Sullivan, P, and L Buske. "Results from CMA's Huge 1998 Physician Survey Point to a Dispirited Profession." *CMAJ*. 1998; 159(5): 525–528.
15. Barbosa GA, Andrade EO, Carneiro MB, Gouveia VV. A saúde dos médicos do Brasil. Brasília: CFM; 2007. 220 p.
16. Bize R, Johnson JA, Plotnikoff RC. Physical activity level and health-related quality of life in the general adult population: a systematic review. Prev Med. 2007; 45(6): 401-15.

17. Pucci GCMF, Rech CR, Fermino RC, Reis RS. Associação entre atividade física e qualidade de vida em adultos. Rev Saúde Pública. 2012; 46: 166–79.

18. Stella F, Gobbi S, Corazza, DI, & Costa, JLR. Depressão no idoso: diagnóstico, tratamento e benefícios da atividade física. Motriz. 2002;8(3), 91-98.

19. Lee, I-Min et al. Impact of Physical Inactivity on the World's Major Non-Communicable Diseases. Lancet. 2012; 380(9838): 219–229.

20. Rothon C, Edwards P, Bhui K, Viner RM, Taylor S, Stansfeld SA. Physical activity and depressive symptoms in adolescents: a prospective study. BMC Med. 2010; 8: 32-40.

21. Bitonte RA, DeSanto DJ. Mandatory physical exercise for the prevention of mental illness in medical students. Ment Illn. Sept. 2, 2014; 6(2): 5549-5550.

22. Cheik NC, Reis IT, Heredia RAG, Ventura MDL, Tufik, S, Antunes HKM, & Mello MD. Efeitos do exercício físico e da atividade física na depressão e ansiedade em indivíduos idosos. Revista Brasileira de Ciência e Movimento. 2003;11(3), 45-52.

23. Wolin KY, Yan Y, Colditz GA, Lee IM. Physical activity and colon cancer prevention: a meta-analysis. Br J Cancer. 2009; 100: 611-6.

24. Friedenreich CM. Physical activity and breast cancer: review of the epidemiologic evidence and biologic mechanisms. Recent Results Cancer Res. 2011; 188: 125-39.

25. Pojednic R, Frates E. A parallel curriculum in lifestyle medicine. Clin Teach. Dez. 6, 2015;12: 1–5.

26. Stoutenberg M, Stasi S, Stamatakis E, Danek D, Dufour T, Trilk JL, et al. Physical activity training in US medical schools: Preparing future physicians to engage in primary prevention. Phys Sportsmed. Nov. 2015; 43(4): 388–94.

27. The WHOQOL Group. Development of the World Health Organization WHOQOL-BREF quality of life assessment. Psychol Med. 1998; 28(3): 551-8.

28. Fleck MPA, Louzada S, Xavier M, Chachamovich E, Vieira G, Santos L, et al. Aplicação da versão em português do instrumento abreviado de avaliação da qualidade de vida "WHOQOL-Bref". Rev Saúde Pública. 2000; 34(2): 178-83.

29. Hassed C, de Lisle S, Sullivan G, Pier C. Enhancing the health of medical students: outcomes of an integrated mindfulness and lifestyle program. Adv Health Sci Educ Theory Pract. 2009; 14(3): 387-98.

30. Alves JGB et al. Qualidade de vida em estudantes de Medicina no início e final do curso: avaliação pelo Whoqol-bref. Rev. Bras. Educ. Med. 2010; 34(1): 91-96.
